# Tetrandrine inhibits aldosterone synthesis by covalently targeting CYP11A1 to attenuate hypertension

**DOI:** 10.3389/fphar.2024.1387756

**Published:** 2024-06-14

**Authors:** Simeng Chu, Wei Yang, Yujie Lu, Junjie Li, Jiamin Peng, Wenjuan Liu, Min Jiang, Gang Bai

**Affiliations:** ^1^ State Key Laboratory of Medicinal Chemical Biology, College of Pharmacy and Tianjin Key Laboratory of Molecular Drug Research, Nankai University, Tianjin, China; ^2^ College of Life Health, Dalian University, Dalian, China

**Keywords:** Tetrandrine, CYP11A1, covalent inhibitor, aldosterone, antihypertensive

## Abstract

**Introduction:**

Tetrandrine (Tet) is the main pharmacological component of *Stephania tetrandra* S. Moore, which is a well-documented traditional Chinese medicine known for its diuretic and antihypertensive properties. Unraveling the specific targets and mechanisms of Tet involved in inducing diuresis and mitigating hypertension can provide valuable insights into its therapeutic effects. This study aimed to explore the diuretic and antihypertensive targets and mechanisms of Tet using chemical biology coupled with activity analyses *in vivo* and *in vitro*.

**Methods:**

The diuretic effects of Tet were evaluated using a water-loaded mouse model. The direct target proteins for the diuretic and antihypertensive effects of Tet were determined using chemical biology. Furthermore, the molecular mechanism of Tet binding to target proteins was analyzed using a multidisciplinary approach based on the structure and function of the proteins. Finally, the effects of the Tet-targeted protein on downstream signaling pathways and blood pressure were evaluated in hypertensive model rats.

**Results:**

Tet exhibited significant antihypertensive and potassium-preserving diuretic effects. The mechanism underlying these effects involves the modulation of the enzyme activity by covalent binding of Tet to Cys423 of CYP11A1. This interaction alters the stability of heme within CYP11A1, subsequently impeding electron transfer and inhibiting aldosterone biosynthesis.

**Discussion:**

This study not only revealed the mechanism of the diuretic and antihypertensive effects of Tet but also discovered a novel covalent inhibitor of CYP11A1. These findings contribute significantly to our understanding of the therapeutic potential of Tet and provide a foundation for future research in the development of targeted treatments for hypertension.

## 1 Introduction

Hypertension is considered one of the most important risk factors in the development of cardiovascular diseases and is the one of the primary causes of disability and premature deaths worldwide (Boutouyrie et al., 2021). Aldosterone (ALD) is a vital steroid hormone that regulates the body’s water–mineral balance and blood pressure; it is also a key component of the renin–angiotensin–aldosterone system (Te Riet et al., 2015). An increase in the plasma ALD concentration is the most common cause of hypertension. Prolonged high levels of ALD not only increase circulating blood volume and blood pressure but also stimulate the proliferation of myocardial fibrocytes, causing cardiac hypertrophy, myocardial fibrosis, and arrhythmias (Buffolo et al., 2022; Parksook and Williams, 2023). Therefore, blocking the interactions between ALD and mineralocorticoid receptors (MRs) has become a common approach for treating hypertension (Leopold and Ingelfinger, 2023). MR antagonists, such as spironolactone (Spi) and eplerenone, are well-recognized medications in the clinical management of hypertension. However, the efficacies of these drugs are hampered by their serious hormonal side effects and inability to effectively inhibit plasma ALD levels in daily clinical practice (Lainscak et al., 2015).

Nevertheless, blocking ALD synthesis, which can directly reduce the plasma ALD level and prevent hormonal side effects, is a new direction for the treatment of hypertension (Carey et al., 2022). CYP11A1 encoding the cholesterol side-chain cleavage (cytochrome P450scc) is a key rate-limiting enzyme in ALD biosynthesis. Studies have shown that knocking out CYP11A1 leads to significant decreases in serum ALD levels (Hu et al., 2000; Zhao et al., 2001). Therefore, CYP11A1 is a promising target for the treatment of hypertension.

Fangji (root of *Stephania tetrandra* S. Moore; Menispermaceae) is a traditional Chinese medicine that is used as a diuretic, an antihypertensive, and an antirheumatic agent (Jiang et al., 2020). Tetrandrine (Tet) is a natural bisbenzylisoquinoline alkaloid recognized as the main effective component of Fangji and has been approved in China for decades for treating patients with silicosis, autoimmune diseases, cardiovascular diseases, and hypertension (Bhagya and Chandrashekar, 2016). Remarkably, our previous research results showed that Tet exhibits the same diuretic effects as Fangji extract at comparable pharmaceutical quantities while also demonstrating notable sodium-excreting and potassium-retaining qualities (Supplementary Figures S1-S3). These results indicate that its mechanism of action may be related to that of an ALD antagonist. However, the direct targets and mechanisms of action remain unclear. Therefore, this study aimed to investigate the antihypertensive targets and mechanisms of action of Tet using chemical biology coupled with its activity analyses *in vivo* and *in vitro*.

## 2 Materials and methods

### 2.1 Reagents

Tet (purity ≥98.0%) was purchased from Chengdu Alfa Biotechnology Co., Ltd. (Chengdu, Sichuan, China). Adrenocorticotropic hormone (ATCH, purity ≥98%) was purchased from Shyuanye Co., Ltd. (Shanghai, China). Angiotensin II (Ang II, purity ≥98%) was purchased from Sangon Biotech Co., Ltd. (Shanghai, China). Spironolactone (Spi, purity ≥98%) was purchased from Aladdin (Beijing, China). Potassium (K^+^), sodium (Na^+^), and chloride (Cl^−^) assay kits were purchased from Nanjing Jiancheng Bioengineering Institute (Nanjing, China). The ELISA kits for pregnenolone (Preg), cortisol (Cort), and ALD were obtained from J&L Biotechnology Co., Ltd. (Shanghai, China). Tris(2-carboxyethyl) phosphine hydrochloride (TCEP) and tris(benzyltriazolylmethyl) amine (TBTA) were purchased from Meilunbio (Dalian, China). The CYP11A1 antibody (bs-10099R) and *CYP11A1 siRNA* (h) were obtained from Bioss Co., Ltd. (Beijing, China) and Santa Cruz Biotechnology, Inc. (Santa Cruz, United States), respectively. The plasmids pCMV3-CYP11A1 (HG14886-UT) and pCMV3-adrenodoxin/FDX1 (HG19339-UT) were purchased from Sinobiological Co., Ltd. (Beijing, China).

### 2.2 Cell lines

The human adrenocortical carcinoma cell line (NCI-H295R, CL-0399) and human embryonic kidney 293T (HEK 293T) cells were purchased from the American Type Culture Collection (Rockville, MD, United States). The NCI-H295R cells were cultured in Dulbecco’s modified Eagle medium (DMEM) containing ITS-G, 10% fetal bovine serum (FBS), and 1% penicillin/streptomycin; the HEK 293T cells were cultured in DMEM containing 10% FBS and 1% penicillin/streptomycin. All cells were cultured at 37°C in an incubator with 5% CO_2_.

### 2.3 Animal experiments

Male C57BL/6J mice (18–20 g, license number: SCXK (Jing) 2016–0006), male Wistar–Kyoto rats (WKY, 230–250 g, license number: SCXK (Jing) 2016–0006), and male spontaneously hypertensive rats (SHR, 230–250 g, license number: SCXK (Jing) 2016–0006) were purchased from Beijing Charles River Experimental Animal Technology Co., Ltd. (Beijing, China). The animals were housed in filter-top cages in a conventional facility maintained at 21°C with 12/12 h light/dark cycles and unrestricted access to food and water. All animal experiments were performed in accordance with the National Institutes of Health Guide for the Care and Use of Laboratory Animals and approved by the Laboratory Animals Care and Use Committee of Tianjin University of Traditional Chinese Medicine (LAEC2019013; Tianjin, China). Isoflurane (2%) was used as the anesthetic in the experiments.

### 2.4 Water-loaded mouse model

Thirty mice were divided into five groups (n = 6 each), including control, Tet, and Spi (positive control). After 7 days of intragastric (i.g.) administration of Tet (15, 30, and 60 mg/kg/day, suspended in saline solution containing 4% Tween-80) and Spi (20 mg/kg/day), all the mice were administered intraperitoneal (i.p.) injections of 50 μL/g normal saline to establish the water-loaded mouse model. All mice were then placed in metabolic cages for 24 h for urine collection. The urine volumes were recorded, and the urine Na^+^, K^+^, and Cl^−^ levels were detected using kits as per the manufacturer’s instructions. The mouse sera were collected to determine the serum ALD, Preg, Na^+^, K^+^, and Cl^−^ levels.

### 2.5 Targets fishing and in-gel imaging

An alkynyl-Tet probe was synthesized to capture and fluorescently label the targets. The synthesis process and identification results of the Tet probe are detailed in Supplementary Figures S4–S6. Six of the rats were divided into two groups (n = 3 each) and administered Tet (100 mg/kg/day) and Tet probe (100 mg/kg/day) separately. Seven days after intragastric administration, the adrenal glands of both groups of animals were obtained and lysed. The adrenal gland lysates from the two groups (containing 3 mg of total protein) were added to amino magnetic microspheres to enrich the target proteins from the adrenal glands for subsequent Western blot analysis and protein mass spectrometry identification. The covalently bound Tet proteins in the adrenal glands were detected by an in-gel imaging method, as described in our previous study (Liu et al., 2022).

### 2.6 Immunofluorescence colocalization

BDP TMR azide fluorescent dye (1 μmol/L) and catalyzer (containing CuSO_4_: TCEP: TBTA = 5:5:1) solution were added to the adrenal gland slices obtained from the Tet or Tet probe rats (Section 2.5) and incubated at 37°C for 1 h. Subsequently, the samples were washed with PBST three times, and CYP11A1 (1:500) and IgG H&L (Alexa Fluor^®^ 488; 1:500) antibodies were added for CYP11A1 staining. The slices were next examined under a laser scanning confocal microscope (SP8; Leica, Germany) at excitation/emission (Ex/Em) wavelengths of 488/505 nm for CYP11A1 and 561/633 nm for the Tet probe.

The NCI-H295R cells were cultured in confocal dishes and divided into three groups as follows: Tet (10 μmol/L), Tet probe (1 μmol/L), and competition (Tet 10 μmol/L + Tet probe 1 μmol/L). After 12 h of treatment, the cells were fixed and fluorescence colocalization analysis was performed as described above.

### 2.7 CYP11A1 enzyme activity detection

The HEK 293T cells were cultured in 6-well plates. After 12 h of incubation, the cells were transfected with 3 µg of pCMV3-CYP11A1 and 1 µg of pCMV3-adrenodoxin/FDX1 (m:m = 3:1) plasmids using Lipofectamine^®^3000. After 10 h of transfection, the cells were treated with 22(*R*)-OH-cholesterol (15 μmol/L) and different concentrations of Tet (0, 0.01, 0.1, 1, 10, and 100 μmol/L) for 48 h, where the Tet was dissolved in DMSO containing 0.1% dilute hydrochloric acid (0.1 mol/L HCl) to prepare the Tet mother solution (0.1 mol/L). Then, the cells were lysed, and the supernatants were collected to assess CYP11A1 expression via Western blotting. The culture media were also collected for the Preg-level assays. The CYP11A1 enzyme activity was inferred from the inhibition rate of Preg production (%).

### 2.8 Transient transfection with CYP11A1 small interfering RNA (siRNA)

The NCI-H295R cells were cultured in six-well plates and transfected with *CYP11A1 siRNA* for 48 h, following which the cell lysates were collected for CYP11A1 interference efficiency assay via Western blotting. Then, the NCI-H295R cells were cultured in 96-well plates and divided into 10 groups as follows: five groups were transfected with *CYP11A1 siRNA,* and the other five groups were cultured in DMEM for 18 h. With the exception of the control group, the other groups were treated with either Ang II (0.1 μmol/L) alone or in combination with different concentrations of Tet [Ang II (0.1 μmol/L) + Tet (0.1, 1.0, and 10 μmol/L)] for 24 h, and the cell supernatants were collected for ALD and Cort level detection according to their kit instructions.

### 2.9 Expression and purification of human CYP11A1

The human CYP11A1 cDNA was cloned and inserted into the pET-28a (+) vector, and a His × 6 tag provided by Sangon Biotech Co., Ltd. (Shanghai, China) was added at the C-terminus. Recombinant wild-type (WT) human CYP11A1 and Cys 423 mutant CYP11A1 protein (C423G, Cys mutated to Gly) were then coexpressed with GroEL/ES in *E. coli*. The expressions were induced by the addition of 0.4 mmol/L isopropyl-1-thio-d-galactopyranoside and 1 mmol/L δ-aminolevulinic acid (δ-ALA), and the culture was incubated for another 16 h at 16°C. Finally, the purification was performed as described previously (Pikuleva et al., 1997).

### 2.10 Carbon monoxide (CO) difference spectra

After the reduced P450 enzyme binds to CO, the protein exhibits a significant absorption peak at approximately 450 nm (Cook et al., 2016). Hence, the effect of Tet on the binding of heme to CYP11A1 was detected using the CO differential spectra. Recombinant CYP11A1 WT (0.3 mg/mL) was incubated with Tet (10 μmol/L) and liver microsomes (0.1 mg/mL) in 50 mmol/L potassium phosphate buffer (PBS, pH 7.4, containing 150 mmol/L of NaCl and 20% of glycerol) for 12 h at 4°C. The samples were reduced using sodium dithionite and exposed to 100% CO for 3 min in an airtight container. Subsequently, the reduced CO complex was scanned to record the CO difference spectra between 400 and 680 nm at 3-nm intervals using an Evolution™ 200 UV–visible spectrophotometer (Thermo Fisher Scientific, Inc., Waltham, MA, United States). The CO difference spectral assay between CYP11A1 WT and C423G mutant protein was performed as above.

### 2.11 In-gel imaging assay

For the preincubated Tet probe experiment, recombinant CYP11A1 protein (WT) was divided into two groups as the Tet probe group (incubated with 10 μmol/L Tet probe and 0.1 mg/mL liver microsomes at 4°C for 12 h) and competitive group (preincubated with 10 μmol/L Tet probe and 0.1 mg/mL liver microsomes at 4°C for 12 h, followed by addition of 100 μmol/L Tet in solution and incubation for another 12 h). Then, BDP TMR Azide Dye (10 μmol/L) was added to the two groups of proteins and imaged at 37°C for 1 h (catalyst: 0.1 mol/L, CuSO_4_: 0.1 mol/L, TCEP: 0.1 mol/L, CuSO_4_: TCEP: TBTA = 5:5:1). Precooled MeOH was then added to this solution, and the samples were washed five times, centrifuged at 6,500 g/min for 5 min to collect the protein, and resolved using 0.5% SDS; next, the sample was heated at 65°C for 10 min for reconstitution, and the proteins were heated at 100°C for 5 min to prepare the samples for the SDS-PAGE. The fluorescence intensity changes were detected at 560 nm using a Tanon 5,200 fully automatic multifunctional imaging system.

For the coincubated Tet probe and Tet experiment, the purified CYP11A1 protein was divided into two groups as the Tet probe group (incubated with 10 μmol/L Tet probe and 0.1 mg/mL liver microsomes at 4°C for 12 h) and competitive group (coincubated with 10 μmol/L Tet probe, 100 μmol/L Tet, and 0.1 mg/mL liver microsomes at 4°C for 12 h). Then, BDP TMR azide dye (10 μmol/L) was added to the two groups of proteins and imaged at 37°C for 1 h (catalyst: 0.1 mol/L, CuSO_4_: 0.1 mol/L, TCEP: 0.1 mol/L, CuSO_4_: TCEP: TBTA = 5:5:1). The remaining steps are the same as those described above.

For the experiment involving Tet probe binding with CYP11A1 WT or CYP11A1 C423G, recombinant CYP11A1 WT or CYP11A1 C423G was incubated with Tet (10 μmol/L) and liver microsomes (0.1 mg/mL) in 50 mmol/L PBS for 12 h at 4°C. Next, the BDP TMR azide fluorescent dye (1 μmol/L) and catalyst (containing CuSO_4_: TCEP: TBTA in the ratio of 5:5:1) solution were added to the proteins and incubated at 37°C for 1 h. The following steps are the same as those described above.

### 2.12 Microscale thermophoresis (MST) assay

The MST assay was performed using purified WT CYP11A1 or CYP11A1 C423G. The proteins were labeled using Monolith NT Protein Labeling Kit RED-NHS. Different concentrations of Tet were then added to each protein, and the binding affinities between CYP11A1 WT or CYP11A1 C423G and Tet were detected using a Monolith NT.115 Blue/Red instrument (NanoTemper Technologies, Munich, Germany) at room temperature with 20% LED and medium MST power. The binding affinities are shown as *K*_D_ values.

### 2.13 SHR model

Thirty SHRs were divided into five groups (n = 6 in each group): model, Tet (15, 30, and 60 mg/kg/day), and Spi (20 mg/kg/day, positive control). Additionally, six WKY rats were randomly selected as the controls. Seven days after i.g. administration, all rats were placed in metabolic cages for 24 h for urine collection. The urine volumes were recorded, and the urine Na^+^, K^+^, and Cl^−^ levels were detected according to their kit instructions. The heart rate (HR), systolic blood pressure (SBP), and diastolic blood pressure (DBP) of the rats were also measured. Thereafter, the rats were anesthetized, and the sera were collected to detect the ALD, Cort, Preg, Na^+^, K^+^, and Cl^−^ levels.

### 2.14 Statistical analysis

Each experiment comprised at least three replicates per condition, and all data are expressed in terms of the means ± SD. The data were analyzed using an unpaired *t*-test or one-way analysis of variance (ANOVA), followed by Dunnett’s *post hoc* test using GraphPad InStat (version 7.0; GraphPad Software, Inc., San Diego, CA, United States). The value *p* < 0.05 was considered to be statistically significant.

## 3 Results

### 3.1 Tet exerts a diuretic effect by reducing serum ALD

**FIGURE 1:**
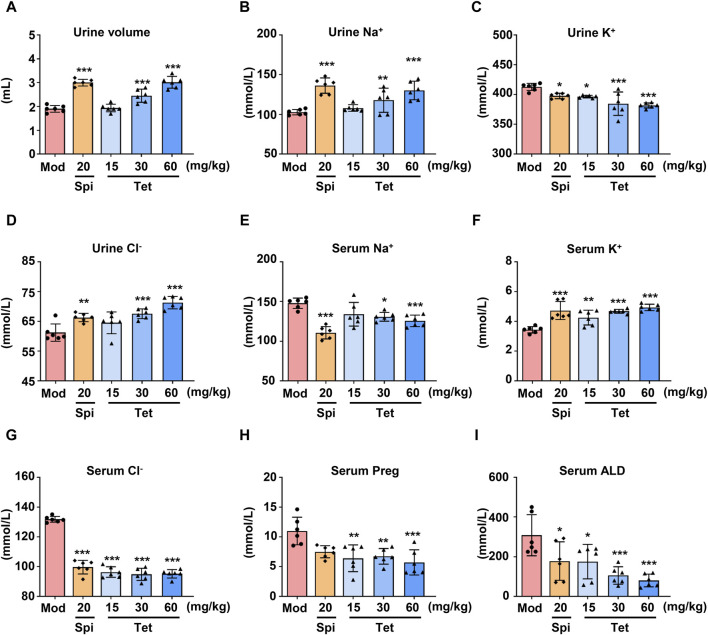
Diuretic effects of Tet in water-loaded mice. **(A)** Urine volume statistics of the water-loaded model mice. **(B**–**G)** Na^+^, K^+^, and Cl^−^ levels in the urine and serum of the model mice analyzed using ion detection kits. **(H,I)** Preg and ALD levels in the serum of the water-loaded mouse model analyzed using ELISA kits. The data are expressed as mean ± SD, n = 6; ^*^ *p* < 0.05, ^**^ *p* < 0.01, ^***^ *p* < 0.001 vs. Mod group.

To study the diuretic effects of Tet, different doses of Tet were administered to the water-loaded model mice, and the urine outputs after administration were measured. As shown in Figure 1A, compared with the model group, the Spi and Tet (30 and 60 mg/kg, respectively) groups had significantly increased urine outputs of the water-loaded mice. Furthermore, the levels of serum Na^+^ and serum Cl^−^ decreased, urine K^+^ decreased, urine Na^+^ and urine Cl^−^ increased, and serum K^+^ increased in the Spi and Tet groups compared with those of the model group (Figures 1B–G). These results indicate that both Spi and Tet exhibit significant diuretic effects by excreting Na^+^ and preserving K^+^. ALD is a mineralocorticoid hormone responsible for regulating the body fluids and ion exchange, and Preg is an important intermediate in the synthesis of this hormone (Johnston et al., 2023). As shown in Figures 1H, I, the serum Preg and ALD levels were reduced in all Tet groups. ALD is synthesized from cholesterol, which is transported to the inner mitochondrial membrane. First, cholesterol is converted to Preg by CYP11A1, then converted to 11-deoxycorticosterone by 3β-hydroxysteroid dehydrogenase and 21-hydroxylase, and finally undergoes hydroxylation and oxidation by aldosterone synthase (CYP11B2) to produce ALD (Williams, 2005). Therefore, we speculate that the diuretic effects of Tet on sodium excretion and potassium retention could be caused by the inhibition of ALD synthesis by Tet.

### 3.2 CYP11A1 in the renal cortex is a target for Tet regulation of ALD

**FIGURE 2:**
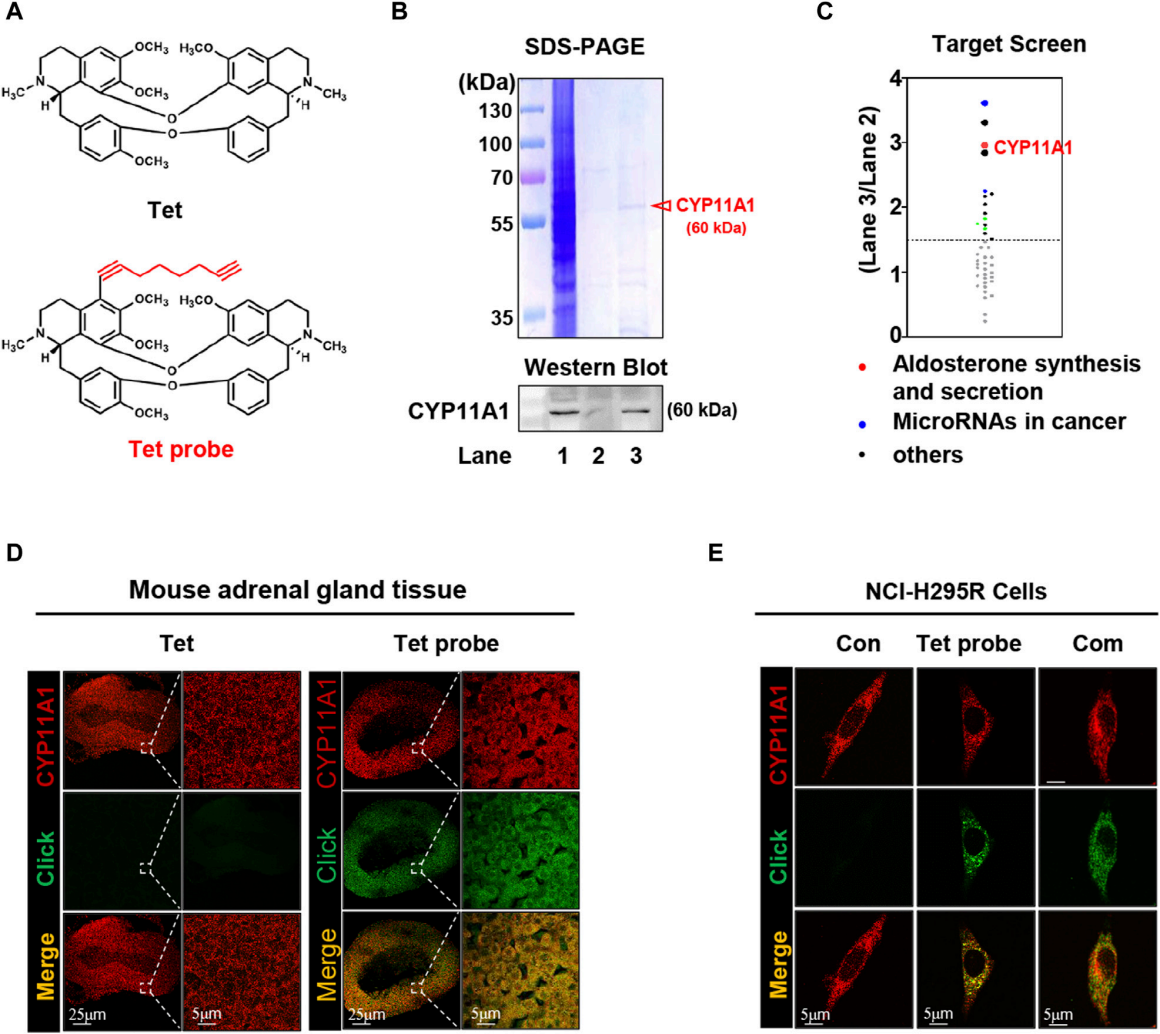
CYP11A1 is identified as the direct target of Tet. **(A)** Chemical structure of Tet and the Tet probe. **(B)** Target identification, including SDS‒PAGE (B, upper panel) and Western blotting (below panel), as well as **(C)** target protein profiling. Lane 1, total protein of the adrenal glands; Lane 2, negative magnetic microspheres; Lane 3, target fishing by the Tet probe. **(D)** Colocalization (pseudo-yellow) of the Tet probe (pseudo-green) and CYP11A1 protein (pseudo-red) in the adrenal gland tissue slices. Tet was used as the control (n = 3). **(E)** Colocalization (pseudo-yellow) of the Tet probe (pseudo-green) and CYP11A1 protein (pseudo-red) in the NCI-H295R cells. The Con group was not treated with drugs; the Tet probe group was administered 1 μmol/L Tet probe, and the Com group was administered 1 μmol/L Tet probe +10 μmol/L Tet (n = 3).

To investigate the diuretic mechanism of Tet, an alkynylation probe (Tet probe) was synthesized and used to capture and fluorescently label the target proteins of Tet based on click reactions with azide (Figure 2A and Supplementary Figure S7). Because the adrenal gland is the site of ALD synthesis, target protein capture was first performed on the adrenal tissue proteins of each group after administering the Tet and Tet probes to the rats. As shown in Figure 2B, compared to the negative control group (Lane 2), the Tet probe group (Lane 3) successfully showed differential protein bands. We identified 41 proteins (score >100) in the bands identified in these two groups using protein mass spectrometry. As shown in Figure 2C, these proteins were sorted according to the score ratios of the two groups (Lane 3/Lane 2), and the results showed 15 proteins with significant differences in their contents (score difference multiple >1.5). Through functional analysis of these 15 differentially expressed proteins using KEGG and validation of the captured proteins using Western blotting (Figure 2B, lower panel), it was preliminarily determined that CYP11A1, which is involved in the synthesis and secretion of ALD, is the diuretic target of Tet.

To visualize the binding of Tet to CYP11A1, we first analyzed its colocalization with CYP11A1 in the adrenal tissue slices using a Tet probe (Supplementary Figures S7). As shown in Figure 2D, compared with the Tet group, the Tet probe group showed specific fluorescence (pseudo-green) in the cortex of the adrenal tissue and colocalized with CYP11A1 (pseudo-red) to emit yellow fluorescence, indicating that Tet binds to CYP11A1 in the cortical area of the adrenal tissue. Subsequently, similar results were observed in the colocalization analysis of the NCI-H295R cells, and the specific pseudo-green fluorescence generated by the Tet probe and yellow fluorescence produced by colocalization with CYP11A1 were competitively weakened by 10 times the amount of Tet (Figure 2E). These results further demonstrate that CYP11A1 is the target of Tet *in vivo* and *in vitro*.

### 3.3 Tet targets CYP11A1 to inhibit ALD synthesis

**FIGURE 3:**
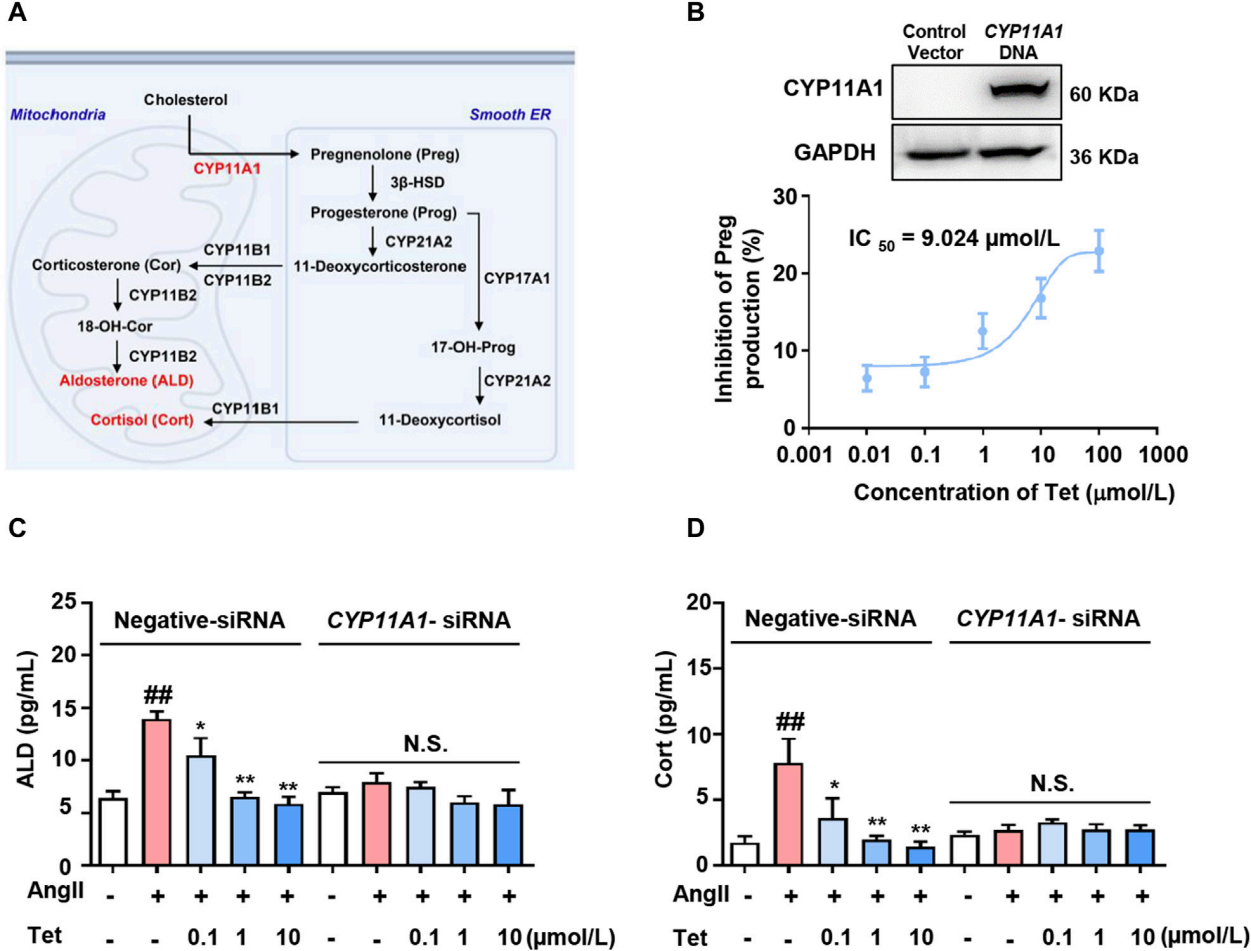
Tet inhibits ALD synthesis by targeting CYP11A1. **(A)** CYP11A1 participates in the ALD and Cort synthesis processes as a key rate-limiting enzyme. **(B)** Western blotting analysis was performed after transfection of the 293T cells with *CYP11A1* DNA for 48 h (n = 3) and CYP11A1 enzyme activity assay of the CYP11A1-overexpressing 293T cells (n = 6). **(C)** ALD and **(D)** Cort level assays in the NCI-H295R cells with *CYP11A1* interference and normal cells (n = 6). The data are expressed as mean ± SD; ^*#*^ *p* < 0.05, ^*##*^ *p* < 0.01, and ^*###*^ *p* < 0.001 vs Con group; ^*^ *p* < 0.05, ^**^ *p* < 0.01, and ^***^ *p* < 0.001 vs. Mod group; N.S., no significant difference.

CYP11A1 is a key rate-limiting enzyme in ALD synthesis and catalyzes the conversion of cholesterol to Preg within the mitochondria (Guo et al., 2003). Subsequently, Preg is transported to the endoplasmic reticulum and converted to 11-deoxycorticosterone and 11-deoxycortisol by a series of enzymes that are catalyzed by ALD and Cort in the mitochondria (Figure 3A). To further evaluate the effects of Tet-targeting of CYP11A1 on its functions, we first overexpressed CYP11A1 in the 293T cells (Figure 3B, upper panel) and next explored the inhibition rate of CYP11A1 by Tet. The results showed that Tet effectively inhibited the catalytic activity of CYP11A1 with an IC_50_ value of 9.024 μmol/L (Figure 3B, lower panel).

To analyze whether the targeted inhibition of CYP11A1 by Tet affects the secretion of adrenal cortical hormones, we stimulated the NCI-H295R cells with Ang II and investigated the effects of Tet on the secretion of ALD and Cort. As shown in Figures 3C, D, under Ang II stimulation, the levels of ALD and Cort secreted by the cells increased significantly, whereas treatment with Tet significantly inhibited the secretion of both these proteins. Moreover, CYP11A1 knockdown completely abolished Tet-mediated inhibition of ALD and Cort production. These results indicate that Tet inhibits ALD synthesis by targeting CYP11A1 activity.

### 3.4 Tet binds to CYP11A1 at the Cys423 site

**FIGURE 4:**
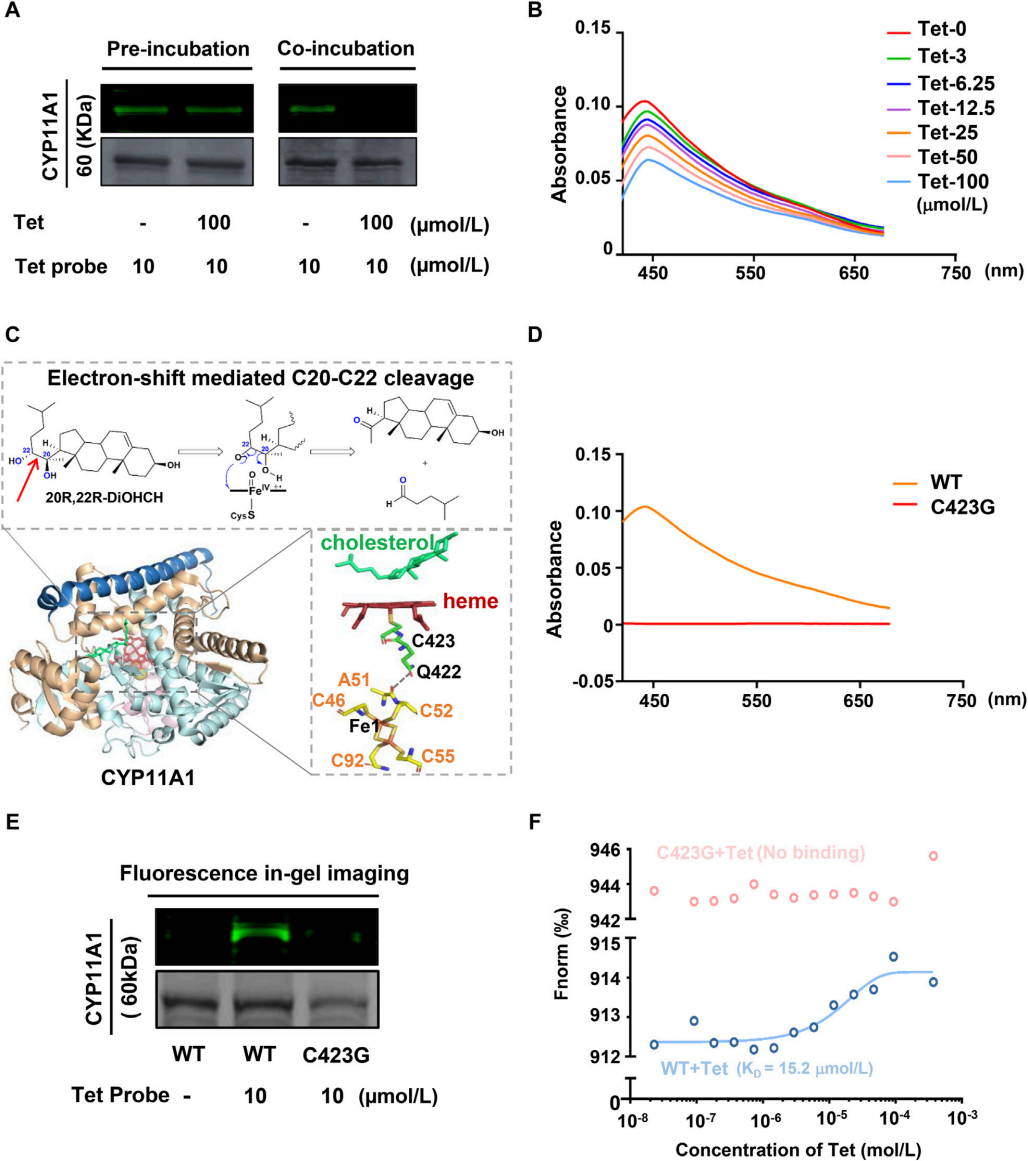
CYP11A1 C423 is identified as the key site for Tet binding. **(A)** Irreversible binding of the Tet probe to CYP11A1. In the preincubation group, the recombinant CYP11A1 protein was preincubated with the Tet probe for 12 h and then incubated with Tet for another 12 h for competitive binding. In the coincubation group, the recombinant CYP11A1 protein was incubated for 12 h with the Tet probe and Tet for competitive binding. **(B)** CO difference spectra analyses of Tet and CYP11A1; the recombinant CYP11A1 protein (0.1 mg/mL) was incubated with Tet (0–100 μmol/L), and the products were reduced with sodium dithionite and complexed with CO. The reduced CO complex was scanned between 400 and 700 nm. **(C)** Schematic of the CYP11A1 catalytic mechanism. **(D)** CO difference spectra between CYP11A1 and C423G. **(E)** Fluorescence in-gel imaging assay between the Tet probe and CYP11A1 or CYP11A1 mutant C423G; **(F)** MST assay of Tet with CYP11A1 or CYP11A1 mutant C423G.

CYP11A1 belongs to the cytochrome P450 enzyme, which is a superfamily containing ferrous heme. The binding of the heme located at the active center to CO yields the characteristic absorption peak of CYP11A1 at 450 nm (Luthra et al., 2011). To verify whether Tet affects this property of CYP11A1, we purified the recombinant CYP11A1 protein (Supplementary Figures S8). As shown in Figure 4A, when CYP11A1 was preincubated with the Tet probe, post-treatment with an excess amount of Tet could not prevent the BDP-Tet probe-labeled CYP11A1 protein. However, the fluorescence intensity of the BDP-Tet probe-labeled CYP11A1 protein was attenuated by coincubation with Tet probe and Tet, meaning that Tet may be covalently bound to CYP11A1. The CO difference spectra analysis showed that Tet disrupted the characteristic absorption peak of CYP11A1 at 450 nm in a concentration-dependent manner (Figure 4B), indicating that Tet-targeting of CYP11A1 could affect the stability of the heme in CYP11A1. The role of the heme in CYP11A1 is mainly to receive electrons from the adrenodoxin (Adx)-containing [2Fe–2S], which then participates in the cholesterol side-chain cleavage reaction (Strushkevich et al., 2011; Slominski et al., 2015). As shown in Figure 4C, electrons are transferred from the Fe1 of the [2Fe–2S] cluster through A51 and C52 in Adx to Q422 of CYP11A1 via spatial jump and are finally transferred to the heme through C423, inducing C20–C22 bond breakage of the cholesterol to produce Preg. The C423 residue of CYP11A1 is also a key amino acid for stabilizing heme (Mast et al., 2011). In our study, we used CO differential spectroscopy to verify binding of C423 to the heme. As shown in Figure 4D, compared with wild-type CYP11A1 protein, the C423 mutant CYP11A1 protein (C423G) did not produce a characteristic absorption peak at 450 nm, indicating that Cys423 is indeed the key amino acid for the CYP11A1 protein to bind to heme. However, Tet not only inhibits the catalytic activity of CYP11A1 but also disrupts the stability of its active center heme in CYP11A1. Therefore, combining the structural and functional analyses of the CYP11A1 protein, we speculate that C423 of CYP11A1 could be the amino acid site at which Tet binds to CYP11A1. Then, fluorescence gel imaging revealed that the Tet probe could successfully label the recombinant human CYP11A1 (WT) but could not fluorescently label the CYP11A1 after site mutation of C423 to G423 (C423G) (Figure 4E). Meanwhile, the MST experimental results showed that Tet could bind to the CYP11A1 protein with a *K*_D_ value of 12.5 μmol/L but not C423G (Figure 4F). These results demonstrate that C423 is the amino acid site where Tet binds to CYP11A1.

### 3.5 Tet exerts antihypertensive effects by targeting CYP11A1 to inhibit ALD production

**FIGURE 5:**
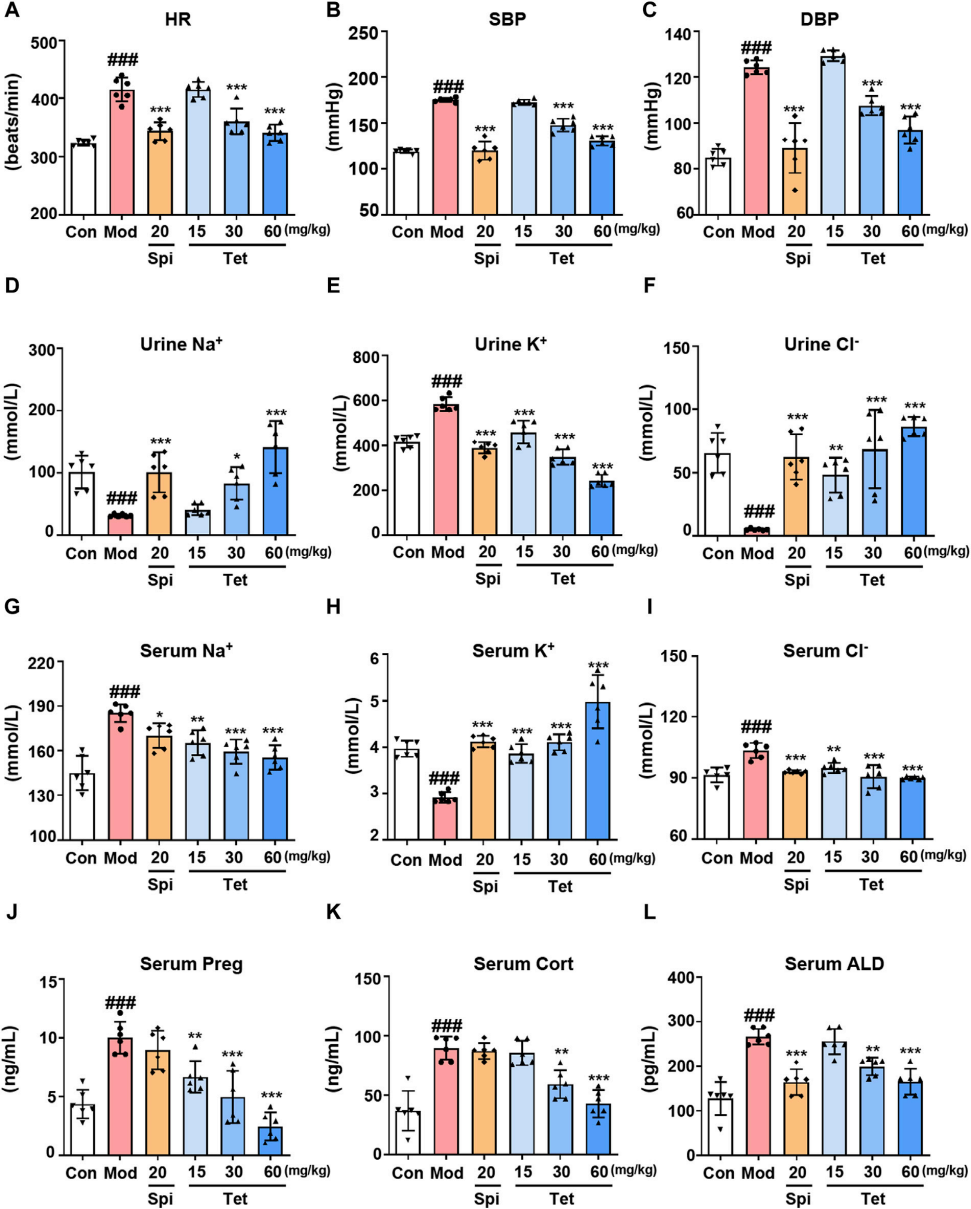
Tet targets CYP11A1 for its antihypertensive effect. **(A–C)** Heart rate as well as systolic and diastolic blood pressures of the SHRs. **(D–I)** Na^+^, K^+^, and Cl^−^ levels in the urine and serum of the SHRs analyzed using ion detection kits. **(J–L)**. The levels of Preg, Cort, and ALD in the serum of the SHRs were analyzed using ELISA kits. The data are expressed as mean ± SD, n = 6; ^*#*^ *p* < 0.05, ^*##*^ *p* < 0.01, and ^*###*^ *p* < 0.001 vs. Con group; ^*^ *p* < 0.05, ^**^ *p* < 0.01, and ^***^ *p* < 0.001 vs. Mod group.

*In vitro* studies have demonstrated that Tet inhibits ALD synthesis by targeting CYP11A1; therefore, we further explored whether Tet exerts antihypertensive effects through this mechanism *in vivo*. After establishing the SHR model and administering different doses of Tet, the blood pressure, serum and urine ion concentrations, and steroid hormone levels were measured. The results show increased HR, SBP, and DBP in the model group compared to those of the control group (Figures 5A–C). In contrast, treatments with Spi (20 mg/kg) and Tet (30 and 60 mg/kg) significantly reduced the HR, SBP, and DBP values in the SHRs. Some important electrolytes in the serum and urine of rats showed that Spi and Tet reduced the levels of serum Na^+^, serum Cl^−^, and urine K^+^ while increasing the levels of urine Na^+^, urine Cl^−^, and serum K^+^ compared to those in the model group (Figures 5D–I). These results confirm that Tet has the effect of “excreting Na^+^ and preserving K^+^.” Serum steroid hormone levels show that Tet significantly reduced serum Preg, Cort, and ALD levels in the SHRs (Figures 5J–L). Although Spi and Tet exhibit similar antihypertensive and ion-changing effects, their mechanisms of action are different. Based on the above results, we found that Tet inhibits production of Preg by targeting CYP11A1 and consequently inhibits its activity, thereby reducing the level of ALD in the body to further regulate the electrolyte balance and ultimately exert an antihypertensive effect.

## 4 Discussion

ALD is a key hormone in the mineralocorticoid pathway that plays fundamental roles in salt and water homeostasis, blood pressure regulation, and cardiovascular remodeling (Parksook and Williams, 2023). Clinical studies have shown that both elevated serum ALD concentration and ALD-to-renin ratio portend a greater risk of developing hypertension (Stowasser, 2021). Angiotensin-converting enzyme inhibitors, Ang II type 1 receptor blockers, and MR antagonists are the commonly used drugs in clinical practice to treat abnormally elevated ALD levels. However, 40%–50% of patients treated with these therapeutics experience return of plasma ALD levels to pretreatment levels over a period of time, accompanied by adverse effects such as tissue remodeling and fibrosis (Bomback and Klemmer, 2007; Mercier et al., 2014). Furthermore, MR antagonists have relatively weaker diuretic and antihypertensive effects than other diuretics. Spi has affinities for progesterone and androgen receptors, which can pose the risk of hyperkalemia and could lead to breast development and impotence in men as well as abnormal menstrual cycles in women (Lainscak et al., 2015). Therefore, the direct inhibition of ALD production to treat diseases related to increased ALD is a promising strategy.

In this study, we investigated the role of CYP11A1 in hypertensive response and demonstrated increased CYP11A1 activity, which is evident from the increased serum Preg, ALD, and Cort levels in the water-loaded mice and SHRs. Furthermore, by employing various chemobiological methods, we validated that Tet, which has antihypertensive effects in clinical practice, significantly reduces hypertension by targeting CYP11A1. These data demonstrate that CYP11A1 is a key regulatory factor in the development of hypertension. Although studies on experimental animal models are difficult to extrapolate to human conditions, identification of the functional significance of CYP11A1 represents a novel and potential target for the treatment of hypertension.

**FIGURE 6:**
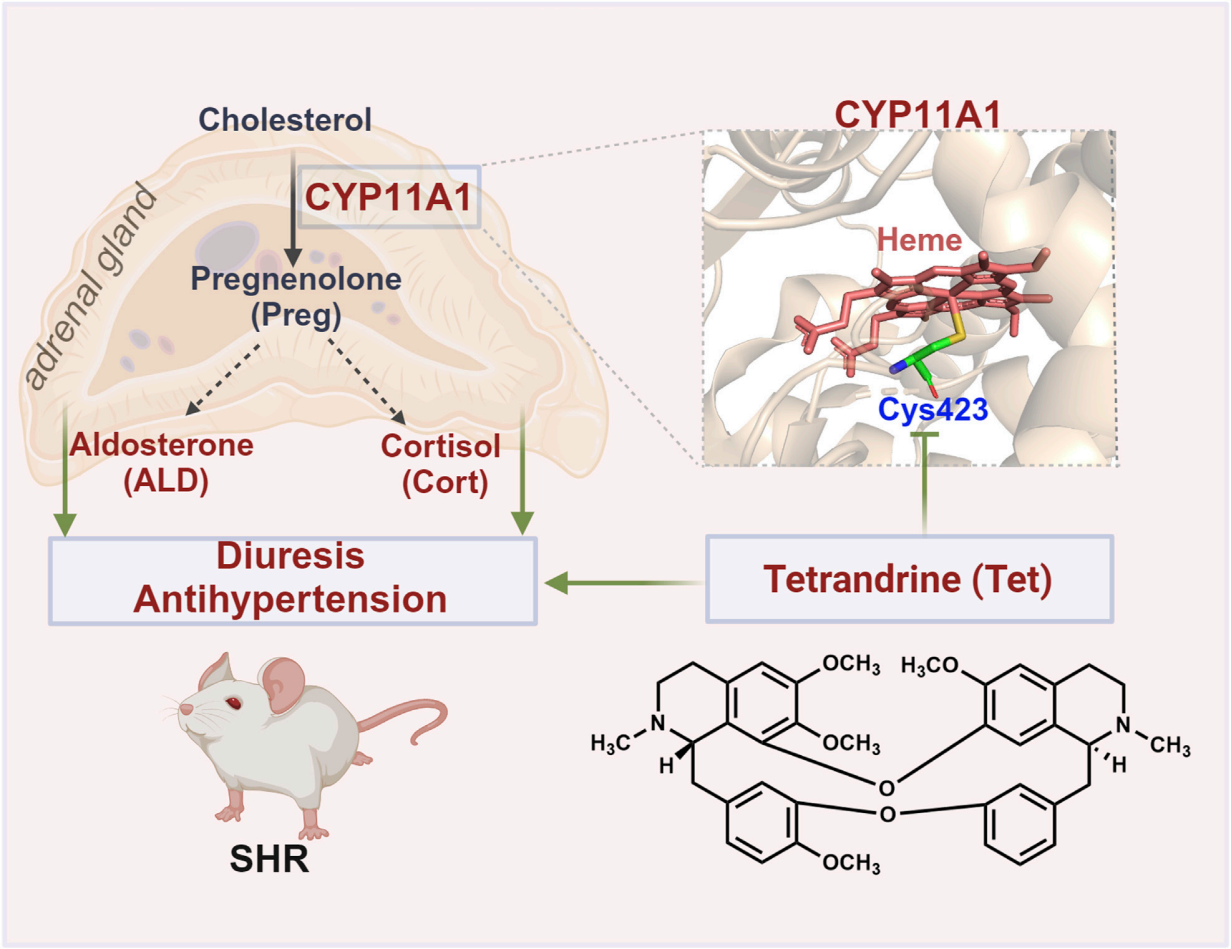
Schematic of the antihypertensive mechanism of Tet targeting CYP11A1. Tet exerts diuretic and antihypertensive effects on the SHRs by covalently binding to the Cys423 site of CYP11A1 to inhibit the biosynthesis of ALD.

CYP11A1 is a mitochondrial monooxygenase and the active center of heme that plays a crucial role in cholesterol cleavage reactions. Currently, CYP11A1 inhibitors such as ketoconazole and posaconazole mostly target the heme. These inhibitors non-covalently bind to the active site of CYP11A1 and coordinate the heme iron with its nitrogen atom, resulting in spectral changes in CYP11A1 and inhibition of its enzymatic activity (Mast et al., 2013). CO differential spectroscopy revealed that Tet induces notable alterations in the characteristic spectral absorption peak of CYP11A1. Tet significantly inhibits the enzyme activity of CYP11A1 with a binding *K*_D_ value of 15.2 μmol/L and IC_50_ value of 9.024 μmol/L. Furthermore, we show that Tet may exert inhibitory enzymatic activity by covalently binding to the Cys423 site of CYP11A1, unlike conventional inhibitors that directly chelate the heme iron. This interaction alters the stability of the heme in CYP11A1 and disrupts electron transfer (Figure 6). Overall, these findings present a novel amino acid site for further development of CYP11A1 inhibitors.

Preg catalyzed by CYP11A1 can be converted to not only ALD but also Cort through a sequence of enzymatic reactions. Cort is a glucocorticoid that increases blood pressure by increasing the blood renin level, regulating the central nervous system, promoting vasoconstriction, and increasing water reabsorption in the renal tubules (Quinkler and Stewart, 2003; Ortiz et al., 2022). Clinical investigations have demonstrated that salivary cortisol levels are elevated in older adults with hypertension (Carstea et al., 2012; Martins et al., 2012). Nevertheless, despite the absence of notable signs or symptoms of Cushing’s syndrome, cardiovascular disease risk is elevated in these patients. These findings suggest that elevated clinical Cort levels also contribute to the complexity of achieving control over hypertension. Together, these results demonstrate the potential of CYP11A1 to simultaneously reduce ALD and Cort, rendering it as a promising candidate for the treatment of hypertension.

Research has shown that steroid hormones play pivotal roles in driving some cancers. CYP11A1 is an important enzyme in the steroid synthesis process, and targeting CYP11A1 has been shown to be an effective strategy for cancer treatment (Kumazawa et al., 2004; Yaspan et al., 2007). AMG is a recognized inhibitor of CYP11A1 enzymatic activity that inhibits adrenal function in patients with Cushing’s syndrome; it is also a second-line therapy for patients with breast and prostate cancers (Samojlik et al., 1980; Santen et al., 2009). ODM-208 is an oral non-steroidal CYP11A1 inhibitor developed by Mercado and Orion, which has progressed to phase II clinical trials for treating metastatic castration-resistant prostate cancers (Karimaa et al., 2022). Furthermore, pharmacological studies have demonstrated the anticancer activities of Tet against prostate, breast, and ovarian cancers (Chen, 2002; Bhagya and Chandrashekar, 2018). However, investigations of the anticancer activities and molecular mechanisms of Tet are still in the initial stages of experimental research and practical use. Therefore, unraveling whether Tet exerts its anticancer effects by targeting CYP11A1 requires further investigation. Meanwhile, the potential risk of hyperkalemia caused by a high dose of Tet should also be monitored in Tet clinical use and optimization. In addition to CYP11A1, Tet could be transferred into methylquinone metabolites by other CYP450s, such as CYP3A5 and CYP2E1, which can cause lung and liver toxicities induced by Tet (Qi et al., 2013; Tian et al., 2016). However, there is no other evidence regarding Tet targeting of CYP450s. Hence, more evidence should be collected to clarify this question. The present study thus provides guidance and direction for further research on Tet, which could be very beneficial in the future.

## 5 Conclusion

This study identifies CYP11A1 as a direct target of Tet for exerting diuretic and antihypertensive effects and unravels the underlying mechanisms by which Tet exerts inhibitory enzyme activity. We demonstrate that Tet covalently binds to the Cys423 site of CYP11A1, thereby altering the stability of the heme in CYP11A1, impeding the electron transfer processes, and inhibiting the biosynthesis of ALD. The findings of this study suggest that CYP11A1 is a promising therapeutic target for hypertension and provides valuable insights for optimizing the development of safer and more effective CYP11A1 inhibitors.

## Data Availability

The original contributions presented in the study are included in the article/Supplementary Material, and any further inquiries may be directed to the corresponding authors.

## Abbreviations

ALD, aldosterone; AMG, aminoglutethimide; Ang II, angiotensin II; ATCH, adrenocorticotropic hormone; Cort, cortisol; Cytochrome P450scc, Cholesterol side-chain cleavage; DBP, diastolic blood pressure; HR, heart rate; MR, mineralocorticoid receptor; Preg, Pregnenolone; SBP, systolic blood pressure; Spi, spironolactone; TBTA, tris-(benzyltriazolylmethyl) amine; TCEP, tris-(2-carboxyethyl) phosphine hydrochloride; Tet probe, alkynylation probe of Tet; Tet, tetrandrine.

## References

[B1] Bhagya N. Chandrashekar K. R. (2016). Tetrandrine - a molecule of wide bioactivity. Phytochemistry 125, 5–13. 10.1016/j.phytochem.2016.02.005 26899361

[B2] Bhagya N. Chandrashekar K. R. (2018). Tetrandrine and cancer - an overview on the molecular approach. Biomed. Pharmacother. 97, 624–632. 10.1016/j.biopha.2017.10.116 29101806

[B3] Bomback A. S. Klemmer P. J. (2007). The incidence and implications of aldosterone breakthrough. Nat. Clin. Pract. Nephrol. 3, 486–492. 10.1038/ncpneph0575 17717561

[B4] Boutouyrie P. Chowienczyk P. Humphrey J. D. Mitchell G. F. (2021). Arterial stiffness and cardiovascular risk in hypertension. Circ. Res. 128, 864–886. 10.1161/CIRCRESAHA.121.318061 33793325

[B5] Buffolo F. Tetti M. Mulatero P. Monticone S. (2022). Aldosterone as a mediator of cardiovascular damage. Hypertension 79, 1899–1911. 10.1161/HYPERTENSIONAHA.122.17964 35766038

[B6] Carey R. M. Moran A. E. Whelton P. K. (2022). Treatment of hypertension: a review. Jama-Journal Am. Med. Assoc. 328, 1849–1861. 10.1001/jama.2022.19590 36346411

[B7] Carstea D. Trasca D. M. Carstea A. P. Trasca E. T. (2012). Study on the dynamics of cortisol secretions in hypertensive elderly patients. Int. J. Hypertens. 2012, 791412. 10.1155/2012/791412 22315668 PMC3270487

[B8] Chen Y. J. (2002). Potential role of tetrandrine in cancer therapy. Acta Pharmacol. Sin. 23, 1102–1106. 12466047

[B9] Cook D. J. Finnigan J. D. Cook K. Black G. W. Charnock S. J. (2016). Cytochromes P450: history, classes, catalytic mechanism, and industrial application. Adv. Protein Chem. Struct. Biol. 105, 105–126. 10.1016/bs.apcsb.2016.07.003 27567486

[B10] Guo I. C. Hu M. C. Chung B. C. (2003). Transcriptional regulation of CYP11A1. J. Biomed. Sci. 10, 593–598. 10.1159/000073524 14576461

[B11] Hu M. C. Chou S. J. Huang Y. Y. Hsu N. C. Li H. Chung B. C. (2000). Tissue-specific, hormonal, and developmental regulation of SCC-LacZ expression in transgenic mice leads to adrenocortical zone characterization (vol 140, pg 5609, 1999). Endocrinology 141, 1235. 10.1210/endo.140.12.7177 10579324

[B12] Jiang Y. P. Liu M. Liu H. T. Liu S. (2020). A critical review: traditional uses, phytochemistry, pharmacology and toxicology of Stephania tetrandra S. Moore (Fen Fang Ji). Phytochem. Rev. 19, 449–489. 10.1007/s11101-020-09673-w 32336965 PMC7180683

[B13] Johnston J. G. Welch A. K. Cain B. D. Sayeski P. P. Gumz M. L. Wingo C. S. (2023). Aldosterone: renal action and physiological effects. Compr. Physiol. 13, 4409–4491. 10.1002/cphy.c190043 36994769 PMC11472823

[B14] Karimaa M. Riikonen R. Kettunen H. Taavitsainen P. Ramela M. Chrusciel M. (2022). First-in-Class small molecule to inhibit CYP11A1 and steroid hormone biosynthesis. Mol. Cancer Ther. 21, 1765–1776. 10.1158/1535-7163.MCT-22-0115 36129801

[B15] Kumazawa T. Tsuchiya N. Wang L. Sato K. Kamoto T. Ogawa O. (2004). Microsatellite polymorphism of steroid hormone synthesis gene CYP11A1 is associated with advanced prostate cancer. Int. J. Cancer 110, 140–144. 10.1002/ijc.20070 15054879

[B16] Lainscak M. Pelliccia F. Rosano G. Vitale C. Schiariti M. Greco C. (2015). Safety profile of mineralocorticoid receptor antagonists: spironolactone and eplerenone. Int. J. Cardiol. 200, 25–29. 10.1016/j.ijcard.2015.05.127 26404748

[B17] Leopold J. A. Ingelfinger J. R. (2023). Aldosterone and treatment-resistant hypertension. N. Engl. J. Med. 388, 464–467. 10.1056/NEJMe2213559 36724334

[B18] Liu W. J. Li Z. Q. Chu S. M. Ma X. Y. Wang X. Y. Jiang M. (2022). Atractylenolide-I covalently binds to CYP11B2, selectively inhibits aldosterone synthesis, and improves hyperaldosteronism. Acta Pharm. Sin. B 12, 135–148. 10.1016/j.apsb.2021.09.013 35127376 PMC8799885

[B19] Luthra A. Denisov I. G. Sligar S. G. (2011). Spectroscopic features of cytochrome P450 reaction intermediates. Arch. Biochem. Biophys. 507, 26–35. 10.1016/j.abb.2010.12.008 21167809 PMC3041835

[B20] Martins L. C. Conceicao F. L. Muxfeldt E. S. Salles G. F. (2012). Prevalence and associated factors of subclinical hypercortisolism in patients with resistant hypertension. J. Hypertens. 30, 967–973. 10.1097/HJH.0b013e3283521484 22406465

[B21] Mast N. Annalora A. J. Lodowski D. T. Palczewski K. Stout C. D. Pikuleva I. A. (2011). Structural basis for three-step sequential catalysis by the cholesterol side chain cleavage enzyme CYP11A1. J. Biol. Chem. 286, 5607–5613. 10.1074/jbc.M110.188433 21159775 PMC3037674

[B22] Mast N. Linger M. Pikuleva I. A. (2013). Inhibition and stimulation of activity of purified recombinant CYP11A1 by therapeutic agents. Mol. Cell Endocrinol. 371, 100–106. 10.1016/j.mce.2012.10.013 23089212 PMC3568244

[B23] Mercier K. Smith H. Biederman J. (2014). Renin-angiotensin-aldosterone system inhibition: overview of the therapeutic use of angiotensin-converting enzyme inhibitors, angiotensin receptor blockers, mineralocorticoid receptor antagonists, and direct renin inhibitors. Prim. Care 41, 765–778. 10.1016/j.pop.2014.08.002 25439533

[B24] Ortiz R. Kluwe B. Lazarus S. Teruel M. N. Joseph J. J. (2022). Cortisol and cardiometabolic disease: a target for advancing health equity. Trends Endocrinol. Metab. 33, 786–797. 10.1016/j.tem.2022.08.002 36266164 PMC9676046

[B25] Parksook W. W. Williams G. H. (2023). Aldosterone and cardiovascular diseases. Cardiovasc. Res. 119, 28–44. 10.1093/cvr/cvac027 35388416

[B26] Pikuleva I. A. Bjorkhem I. Waterman M. R. (1997). Expression, purification, and enzymatic properties of recombinant human cytochrome P450c27 (CYP27). Arch. Biochem. Biophys. 343, 123–130. 10.1006/abbi.1997.0142 9210654

[B27] Qi X. M. Miao L. L. Cai Y. Gong L. K. Ren J. (2013). ROS generated by CYP450, especially CYP2E1, mediate mitochondrial dysfunction induced by tetrandrine in rat hepatocytes. Acta Pharmacol. Sin. 34 (9), 1229–1236. 10.1038/aps.2013.62 23892269 PMC4003161

[B28] Quinkler M. Stewart P. M. (2003). Hypertension and the cortisol-cortisone shuttle. J. Clin. Endocrinol. Metab. 88, 2384–2392. 10.1210/jc.2003-030138 12788832

[B29] Samojlik E. Veldhuis J. D. Wells S. A. Santen R. J. (1980). Preservation of androgen secretion during estrogen suppression with aminoglutethimide in the treatment of metastatic breast carcinoma. J. Clin. Invest. 65, 602–612. 10.1172/JCI109705 6986409 PMC371401

[B30] Santen R. J. Brodie H. Simpson E. R. Siiteri P. K. Brodie A. (2009). History of aromatase: saga of an important biological mediator and therapeutic target. Endocr. Rev. 30, 343–375. 10.1210/er.2008-0016 19389994

[B31] Slominski A. T. Li W. Kim T. K. Semak I. Wang J. Zjawiony J. K. (2015). Novel activities of CYP11A1 and their potential physiological significance. J. Steroid Biochem. Mol. Biol. 151, 25–37. 10.1016/j.jsbmb.2014.11.010 25448732 PMC4757911

[B32] Stowasser M. (2021). Aldosterone and primary aldosteronism: star performers in hypertension research. Hypertension 78, 747–750. 10.1161/HYPERTENSIONAHA.121.17594 34379432

[B33] Strushkevich N. Mackenzie F. Cherkesova T. Grabovec I. Usanov S. Park H. W. (2011). Structural basis for pregnenolone biosynthesis by the mitochondrial monooxygenase system. Proc. Natl. Acad. Sci. U. S. A. 108, 10139–10143. 10.1073/pnas.1019441108 21636783 PMC3121847

[B34] Te Riet L. Van Esch J. H. M. Roks A. J. M. Van Den Meiracker A. H. Danser A. H. J. (2015). Hypertension renin-angiotensin-aldosterone system alterations. Circulation Res. 116, 960–975. 10.1161/CIRCRESAHA.116.303587 25767283

[B35] Tian Y. Shen S. Jiang Y. Shen Q. Zeng S. Zheng J. (2016). CYP3A5 mediates bioactivation and cytotoxicity of tetrandrine. Arch. Toxicol. 90 (7), 1737–1748. 10.1007/s00204-015-1584-8 26302866

[B36] Williams G. H. (2005). Aldosterone biosynthesis, regulation, and classical mechanism of action. Heart Fail Rev. 10, 7–13. 10.1007/s10741-005-2343-3 15947886

[B37] Yaspan B. L. Breyer J. P. Cai Q. Dai Q. Elmore J. B. Amundson I. (2007). Haplotype analysis of CYP11A1 identifies promoter variants associated with breast cancer risk. Cancer Res. 67, 5673–5682. 10.1158/0008-5472.CAN-07-0467 17575134 PMC2805128

[B38] Zhao L. P. Bakke M. Parker K. L. (2001). Pituitary-specific knockout of steroidogenic factor 1. Mol. Cell. Endocrinol. 185, 27–32. 10.1016/s0303-7207(01)00621-9 11738791

