# Supplementary material for: Tetrandrine inhibits aldosterone synthesis by covalently targeting CYP11A1 to attenuate hypertension
Source: Front Pharmacol. 2024 Jun 14;15:1387756. doi: 10.3389/fphar.2024.1387756 (PMC11211567; doi:10.3389/fphar.2024.1387756)

**The origin western blot**

**Figure 1: The original image of Fig 2B in manuscript was presented as follow:**


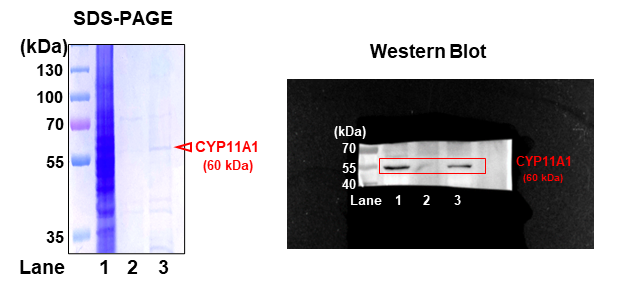


**Figure 2: The original image of Fig 3B for three repeats in manuscript was presented as follow:**


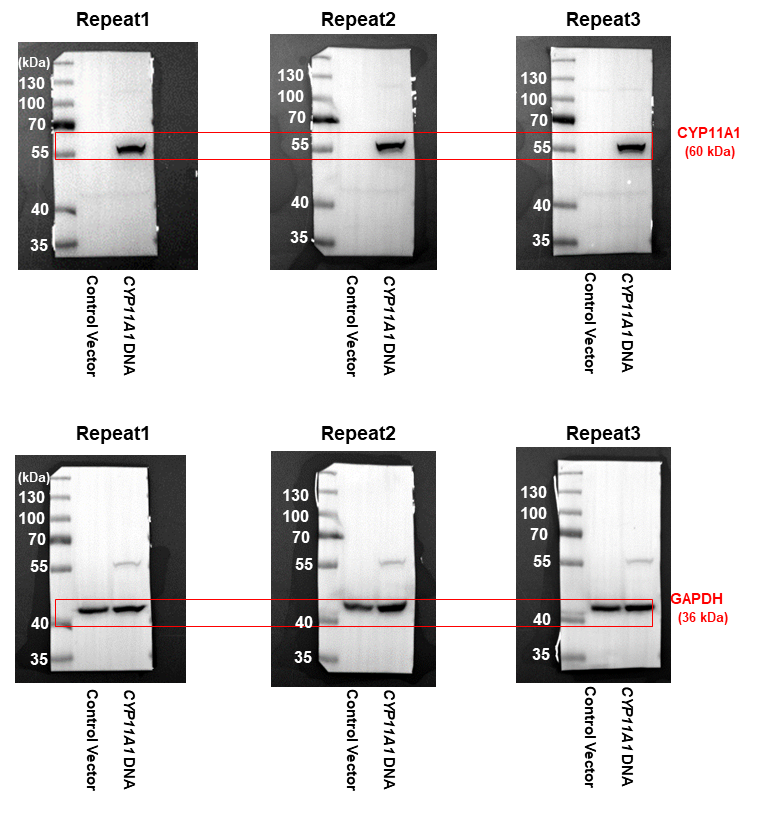


**Figure 3: The original image of Fig 4A in manuscript was presented as follow:**

**
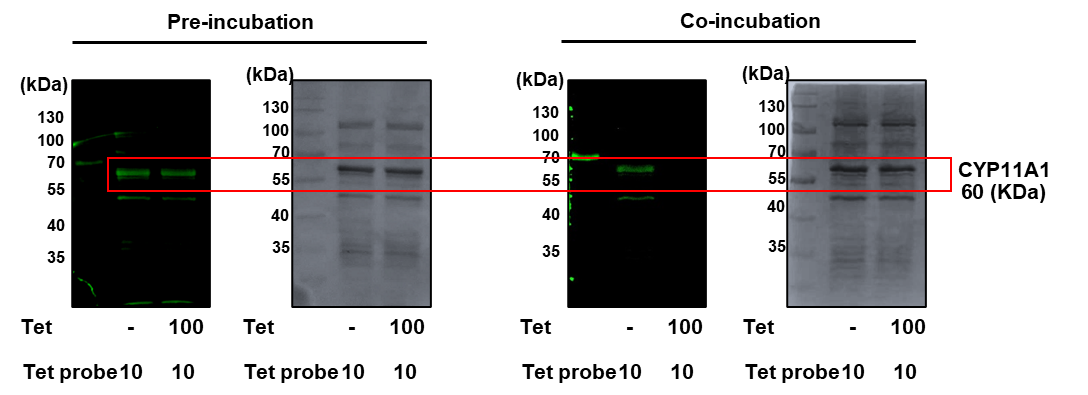
**

**Figure 4: The original image of Fig 4E in manuscript was presented as follow:**


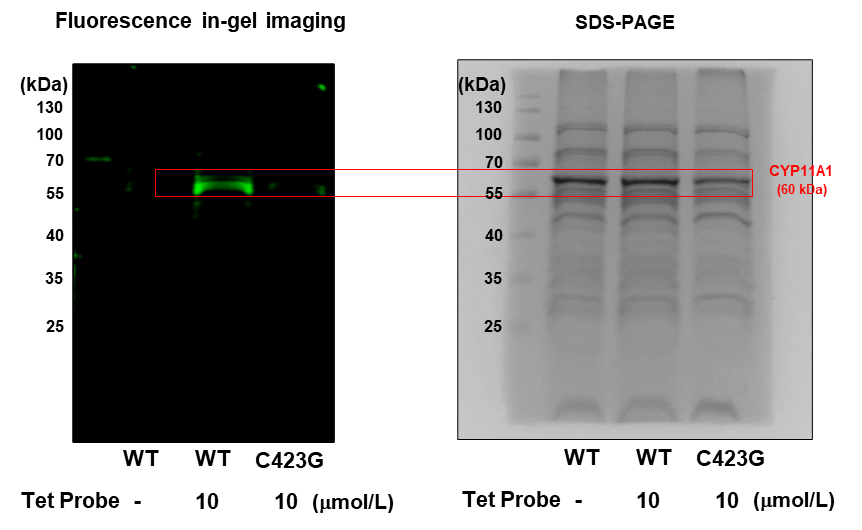

Supplement: Supplementary file 1 [file DataSheet2.docx]
