# Supplementary material for: Tetrandrine inhibits aldosterone synthesis by covalently targeting CYP11A1 to attenuate hypertension
Source: Front Pharmacol. 2024 Jun 14;15:1387756. doi: 10.3389/fphar.2024.1387756 (PMC11211567; doi:10.3389/fphar.2024.1387756)

**Supporting information**

**Tetrandrine inhibits aldosterone synthesis by covalently targeting CYP11A1 to attenuate hypertension**

**Simeng Chu^1^, Wei Yang^1^,** **Yujie Lu^2^, Junjie Li^1^, Jiamin Peng^1^, Wenjuan Liu^1^*, Min Jiang^1^*, Gang Bai^1^**

^1^ State Key Laboratory of Medicinal Chemical Biology, College of Pharmacy and Tianjin Key Laboratory of Molecular Drug Research, Nankai University, Tianjin, 300353, China

^2^ College of Life Health, Dalian University, Dalian, 116622, China

**Correspondence**

Wenjuan Liu, State Key Laboratory of Medicinal Chemical Biology, College of Pharmacy and Tianjin Key Laboratory of Molecular Drug Research, Nankai University, Tianjin, 300353, China.

Email: [liuwenjuan@nankai.edu.cn](mailto:liuwenjuan@nankai.edu.cn);

Min Jiang, State Key Laboratory of Medicinal Chemical Biology, College of Pharmacy and Tianjin Key Laboratory of Molecular Drug Research, Nankai University, Tianjin, 300353, China. Email: [minjiang@nankai.edu.cn](mailto:minjiang@nankai.edu.cn). Phone/Fax: +86-22-23506930.

**1. Determination of Tetrandrine content in Fangji**

**1) Methods:**

***1.1) Materials***

Fangji (the root of *Stephania tetrandra* S. Moore; Menispermaceae) (2202036) used in the current experiment was purchased Beijing Tongrentang Co., Ltd (Beijing, China). Tetrandrine (Tet, purity ≥ 98.0%) was purchased from Chengdu Alfa Biotechnology Co.,Ltd (Chengdu, Sichuan, China).

***1.2) Standard Preparation and Calibration Curve***

The reference compounds Tet was accurately weighed and dissolved in methanol to produce a mixed standard stock solution with the concentrations of 12.5, 25.0, 50.0, 100 and 200 µg/mL respectively for HPLC (SHIMADZU, Japan) analysis to make calibration curves.

***1.3) Sample Preparation***

1.0 g Fangji decoction pieces were extracted with 10 mL water for 1h, concentrated freeze-drying to obtained Fangji extraction freeze-dried powder. Dissolved Fangji power with 5.8 mL methanol and filterated using 0.22 µm filter membrane to prepared sample for HPLC analysis.

***1.4) Chromatographic Conditions***

HPLC condition: chromatographic column: COSMOSIL 5PBR (4.6 × 250 mm, 5 μm); mobile phase: 0.5% Formic acid water (A) – methanol (B), gradient elution (0∼5 min, 10% B ⟶ 10% B; 5–20 min, 10%B ⟶ 80% B; 25–30 min, 80% B ⟶ 80% B; 30–31 min, 80% B ⟶ 10% B; 31–35 min, 10% B ⟶ 10% B); detection wavelength: 282 nm; column temperature: 30°C; current velocity: 1 mL∙min−1; sample size: 10 μL. The compounds were quantified by dividing the peak areas of the compounds of interest by the peak area of the standard compound.

**2) Results:**

***2.1) Standard curve:***

Based on the direct ratio between the concentration of Tet and its absorbance, the standard curve was calculated by different concentrations of Tet and the pear area. The The linear regression equation was y = 3129.1x + 3985.5 (R^2^ = 0.9999) as showed in Fig S1.


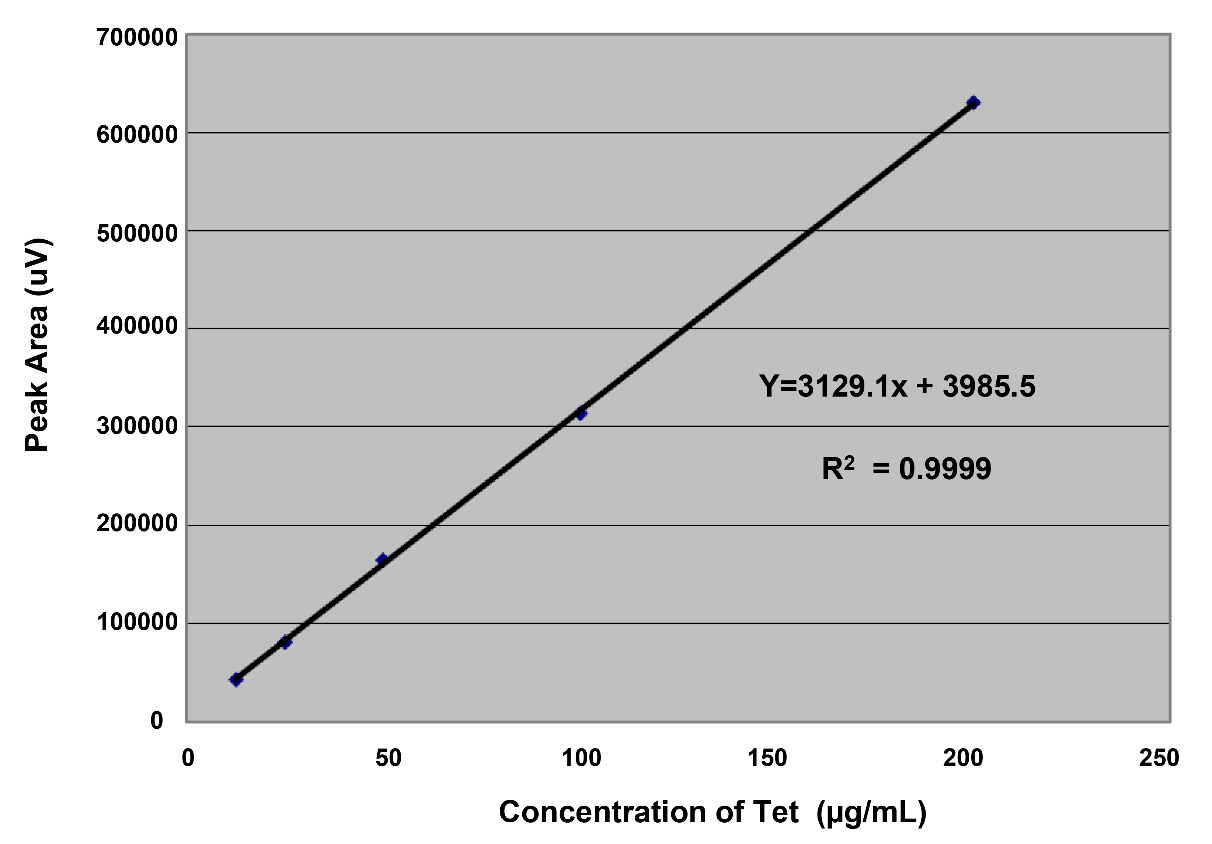


**Fig S1. The standard curve of Tet.**

***2.2) The content of Tet from Fangji***

The Fangji extraction samples (n=3) were analyzed by HPLC and chromatogram was showed as Fig. S2. Then, the content of Tet from Fangji calculated by standard curve was 8.575 mg/g, which mean each gram of Fangji contains 8.575mg of Tet.

**
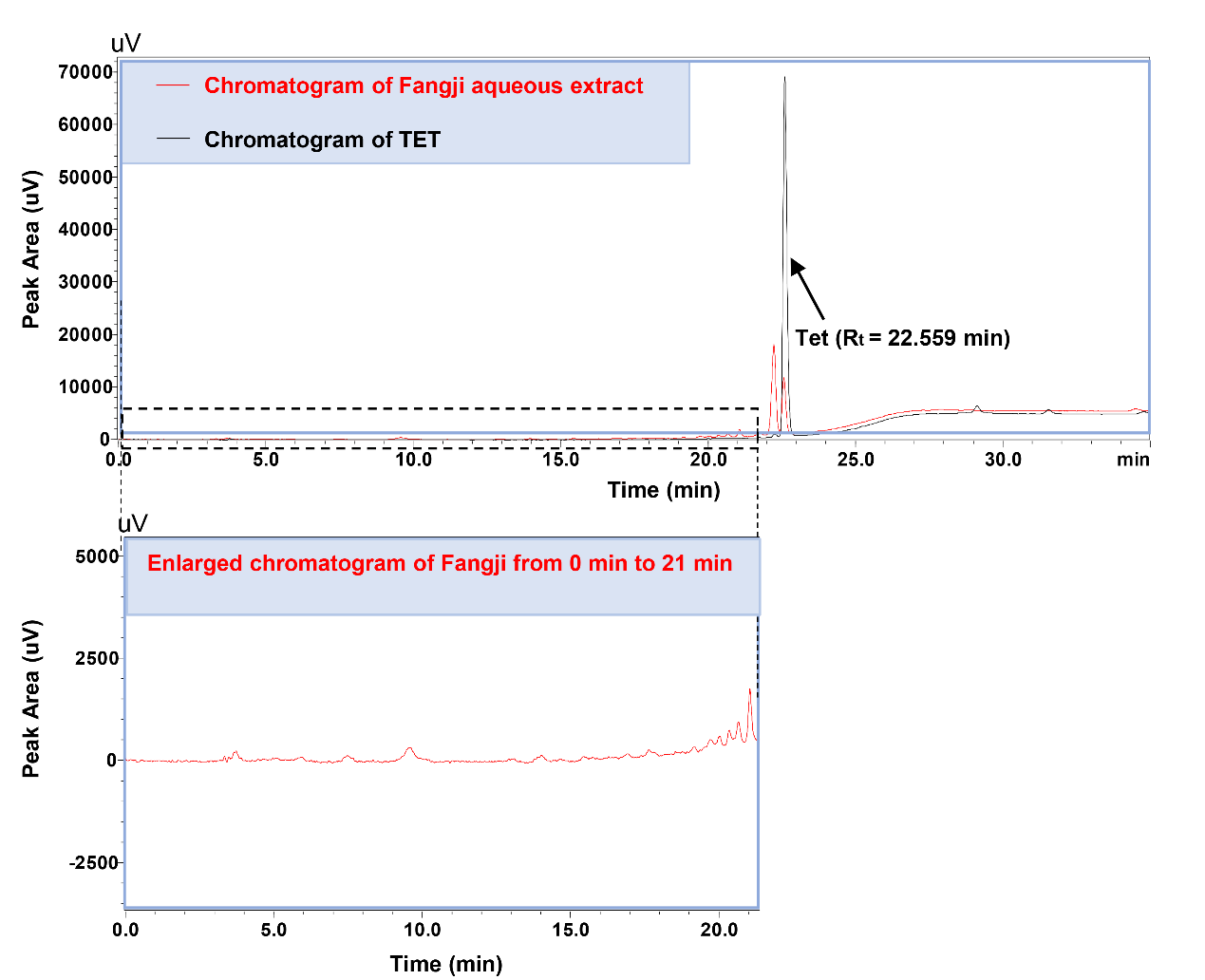
**

**Fig. S2. The chromatogram of Fangji aqueous extract and Tet. The Rt of Tet was 22.559 min.**

**2. Evaluation of diuretic effect of Fangji aqueous extract and Tet**

**1) Methods:**

***1.1) Animal experiments***

Male C57BL/6J mice (18–20 g, license number: SCXK (Jing) 2016–0006) were purchased from Beijing Charles River Experimental Animal Technology Co., Ltd. (Beijing, China). Animals were housed in filter-top cages in a conventional facility maintained at 21 °C with a 12/12 h light/dark cycle and unrestricted access to food and water. All animal experiments were performed in accordance with the National Institutes of Health Guide for the Care and Use of Laboratory Animals and approved by the Tianjin University of Traditional Chinese Medicine Laboratory Animals Care and Use Committee (LAEC2019013; Tianjin, China). Isoflurane (2%) was used as an anesthetic.

***1.2) Water-loaded mice model***

Forty-eights mice were divided into five groups (n = 6 each): control, Fangji aqueous extract, Tet, and Spi (positive control). After 7 days of intragastric (i.g.) administration of Fangji aqueous extract (1.75, 3.5, and 7 g/kg/day), Tet (15, 30, and 60 mg/kg/day) and Spi (20 mg/kg/day), all mice were injected with 50 µL/g normal saline to establish a water-loaded mice model. All mice were then placed in metabolic cages to collect urine for 24 h. Urine volumes were recorded, and urine Na^+^, K^+^, and Cl^−^ levels were detected according to the kit instructions. Mouse serum was collected to determine serum ALD, Preg, Na^+^, K^+^, and Cl^−^ levels.

**2) Results:**

It is calculated that each gram of Fangji contains 8.575mg of Tet, so we compared the diuretic effect of Fangji aqueous extract with the corresponding amount of Tet. The results showed that Tet exhibits the same diuretic effect as Fangji extract at comparable pharmaceutical quantities, and demonstrates a notable sodium-excreting and potassium-retaining phenomenon in terms of the diuretic effect, which may be related to antagonistic serum Preg and ALD (Fig. S3).


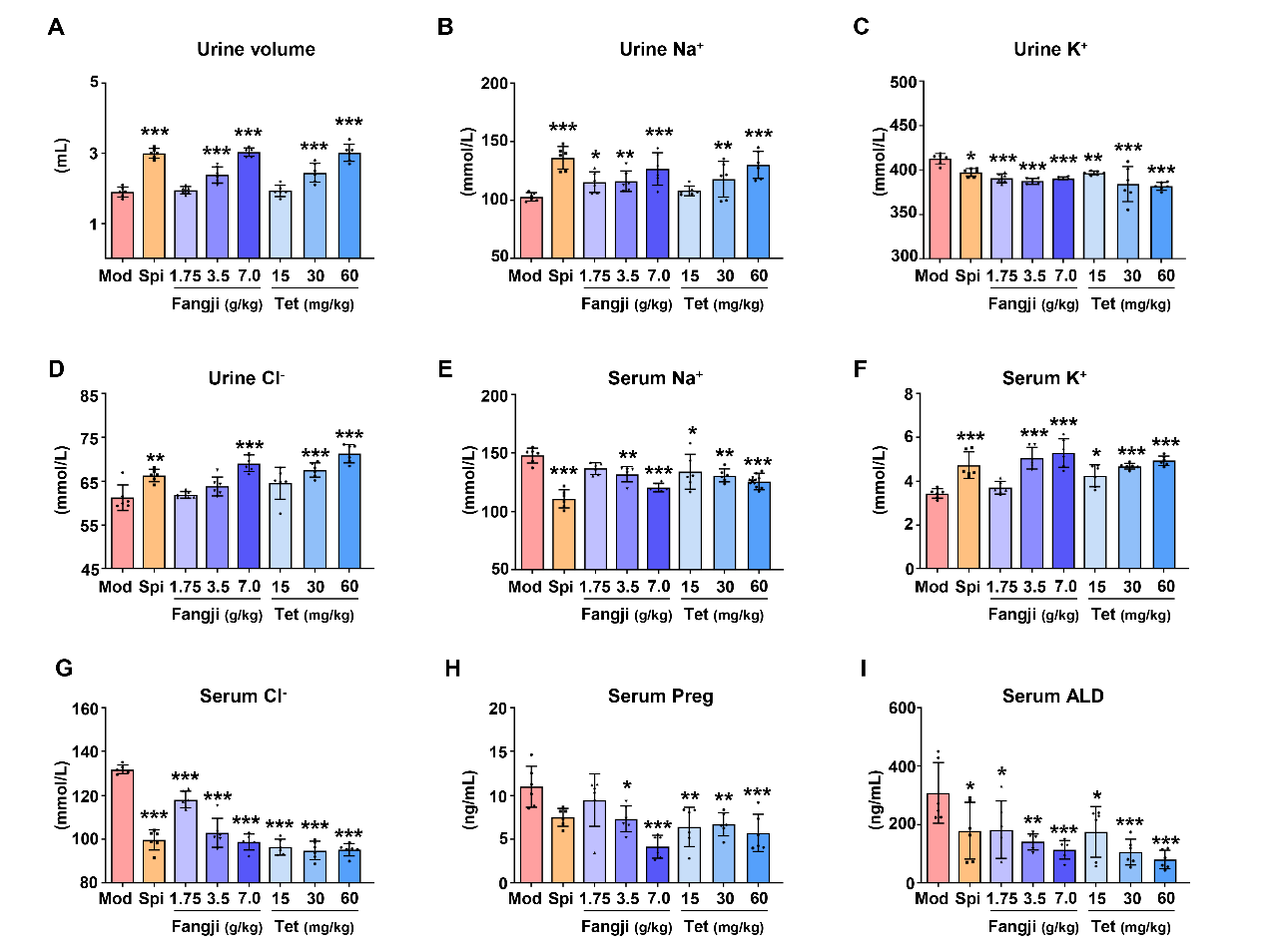


**Fig. S3. Diuretic effect** **of Fangji aqueous extraction and Tet in water-loaded mice.** (A) Urine volume statistics of water-loaded-model mice. (B)-(G) The contents of Na^+^, K^+^, and Cl^−^ in the urine and serum of model mice analyzed using ion detection kits. (H) and (I) The contents of Preg and ALD in the serum of the water-loaded mice model were analyzed by ELISA kits. Data are expressed as the mean ± SD, n = 6; *^*^P* < 0.05, *^**^P* < 0.01, *^***^P* < 0.001 vs. Mod group.

**3.** **Synthetic route of the Tet probe**

AM-TET was synthesized by WUXI Apptec (Wuxi, Jiangsu, China; an integrated R&D Service company) according to the synthetic route in Fig. S4. The hydrogen spectrum and MS spectrum are shown in Fig. S5 and S6, respectively.

**Fig. S4. Synthetic schemes for the Tet probe.**

**The details of synthetic route of the Tet probe:**

**Project Summary**

The synthetic route is based on a procedure supplied by **WuXi**.

**Experimental for largest scale run:**

***General procedure for preparation of compound* *b*** *-* ***Notebook Page: ET55433-8***

A solution of compound **a1** (8.03 g, 79.3 mmol, 11.2 mL, 2.50 eq) in toluene (28 mL) was cooled to 0°C. Then a solution of compound **a** (7.00 g, 31.7 mmol, 5.69 mL, 1.00 eq) in toluene (14 mL) was added under vigorous stirring, and the mixture was warmed to 20°C and stirred for 12 hrs. TLC (Petroleum ether: Ethyl acetate = 5:1, R_f_ = 0.69) indicated the product was detected. The reaction mixture was concentrated under reduced pressure to remove toluene. The residue was diluted with H_2_O (100 mL) and extracted with EtOAc (50 mL, 30 mL). The combined organic layers were washed with brine (40 mL), dried over Na_2_SO_4_, filtered and concentrated under reduced pressure to give a residue. The residue was purified by column chromatography (SiO_2_, Petroleum ether/Ethyl acetate=1/0 to 10/1). Compound **b** (5.20 g, 18.2 mmol, 57.4% yield) was obtained as a white solid.

***General procedure for preparation of compound 2 -Notebook Page: ET52615-56***

To a solution of Tetrandrine (10.0 g, 16.1 mmol, 1.00 eq) in H_2_O (12.5 mL) and TFA (25 mL) was added Br_2_ (2.69 g, 16.9 mmol, 869 uL, 1.05 eq) in CH_3_COOH (10 mL). The mixture was stirred at -15-0°C for 4 hrs. LC-MS showed 92% of desired compound was detected. The precipitate was filtered off and the organiclayer washed with water (2 × 25 mL) and dried over Na_2_SO_4_. The drying agent was filtered off and the solvent evaporated atreduced pressure. The residue was purified by column chromatography (SiO_2_, DCM: MeOH = 100/1 to 30/1). Compound **2** (10.5 g, 15.0 mmol, 93.2% yield) was obtained as a light white solid.

***General procedure for preparation of Target - Notebook Page: ET57256-5***

Four reactions were carried out in parallel.

To a solution of compound **2a** (1.51 g, 14.3 mmol, 2.00 eq), compound **2** (5.00 g, 7.13 mmol, 1.00 eq), N, N-diisopropyl-1,1-diphenylphosphanamine (915 mg, 3.21 mmol, 0.45 eq), K_2_CO_3_ (2.95 g, 21.4mmol, 3.00 eq), diacetoxypalladium (240 mg, 1.07 mmol, 0.15 eq) in THF (10 mL). The mixture was stirred at 65°C for 12 hrs. LC-MS showed 35% of compound **2a** remained. Several new peaks were shown on LC-MS and 43% of desired compound was detected.  The reaction mixture was diluted with H_2_O (150 mL) and extracted with EtOAc (100 mL, 50 mL). The combined organic layerswere washed with brine (50.0 mL), dried over Na_2_SO_4_, filtered and concentrated under reduced pressure to give a residue. The residue of four reactions was purified by prep-HPLC (column: Phenomenex luna C18 (250*70mm,10 µm); mobile phase: [water (TFA)-ACN]; B%: 10%-50%, 20 min) to give **Target** (2.1 g, 2.88 mmol, 9.86% yield) as a yellow solid.


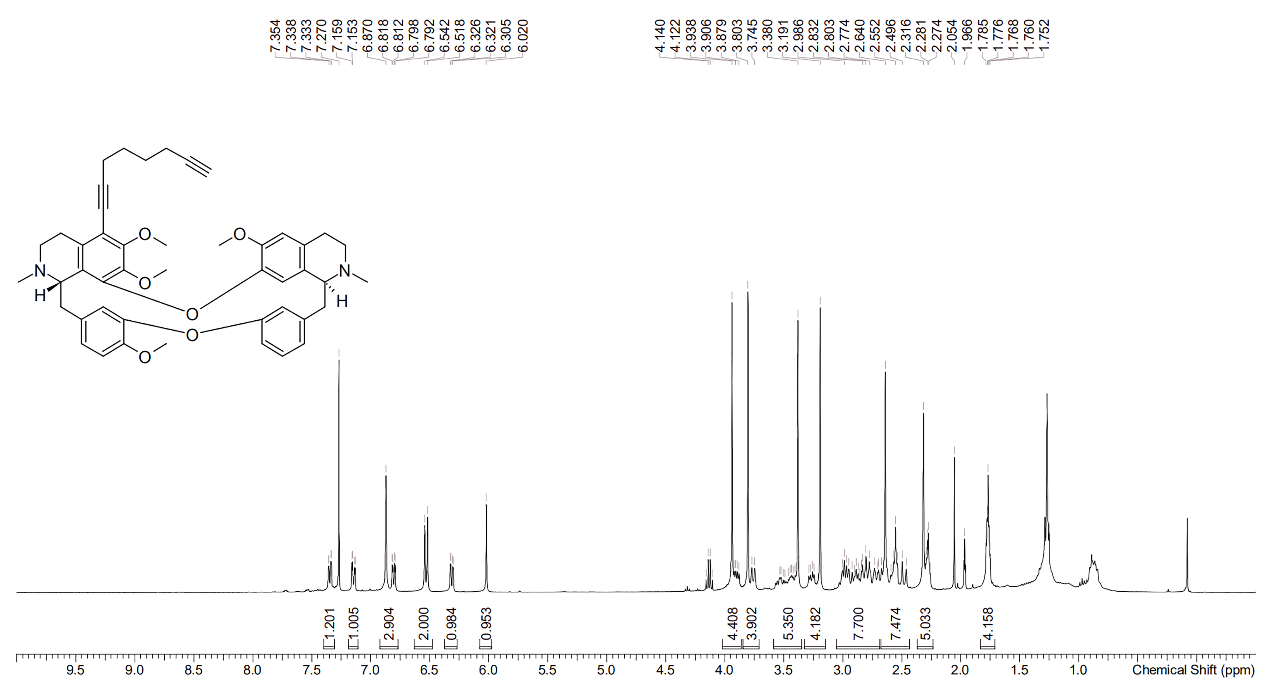


**Fig. S5. Hydrogen spectrum of the Tet probe.**


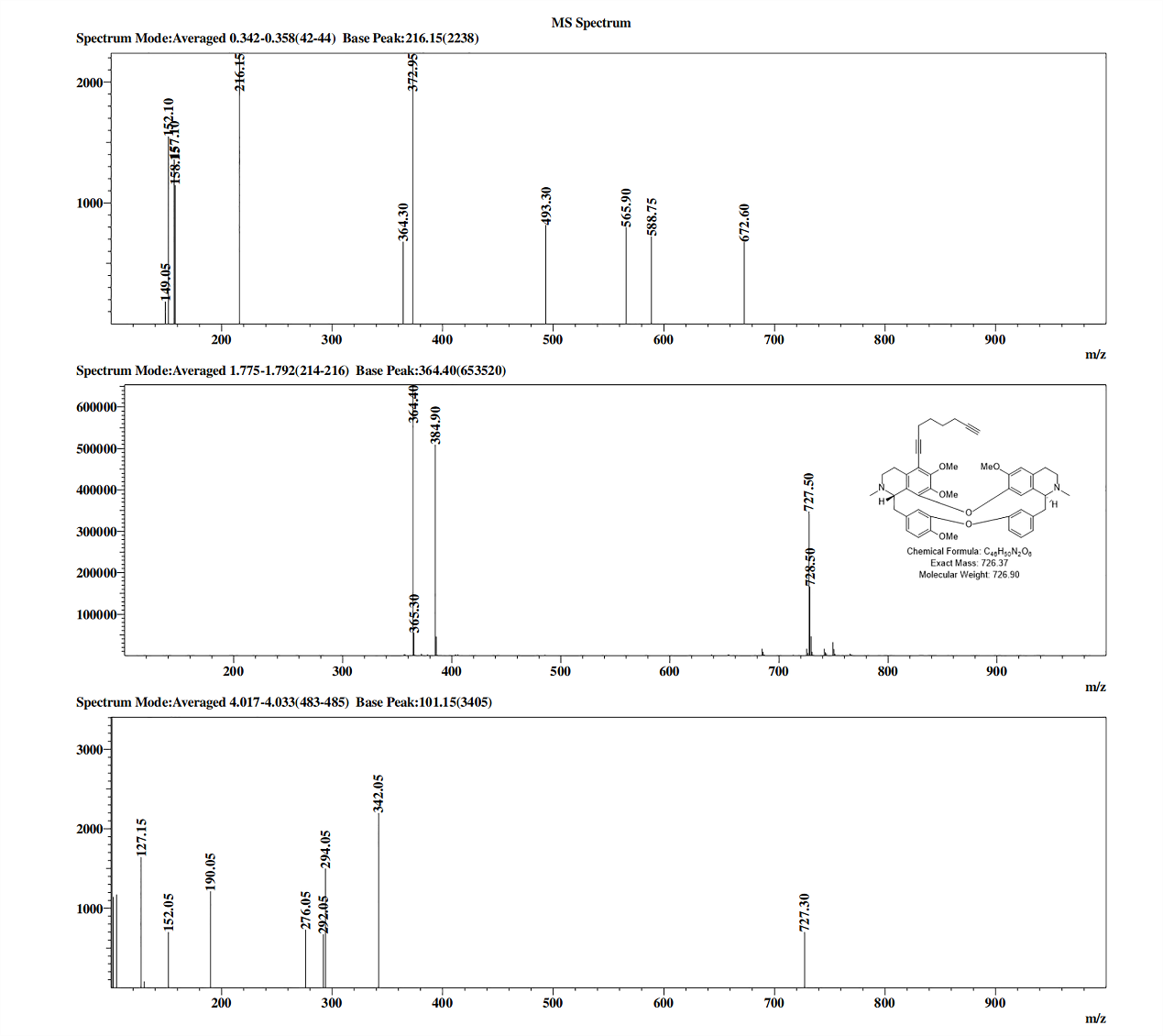


**Fig. S6. MS spectrum of the Tet probe.**

**LCMS method of Tet probe:**

| Method name: | 5_95AB_6min-220 | | | | | | |
| --- | --- | --- | --- | --- | --- | --- | --- |
| Instrument: | Agilent 1200 & 6110B | | | | | | |
| Column: | Luna-C18(2) 2.0*50mm,5um | | | | | | |
| Column temperature: | 40 °C | | | | | | |
| Mobile phase A(MPA) | H_2_O+0.04 %(v/v) TFA | | | | | | |
| Mobile phase B(MPB) | ACN+0.02 %(v/v) TFA | | | | | | |
| Flow rate: | 1.0 mL/min | | | | | | |
| Gradient Ratio: | Time(min) | 0.00 | 0.40 | 3.00 | 4.00 | 4.01 | 4.50 |
|  | MPA (%) | 95 | 95 | 5 | 5 | 95 | 95 |
|  | MPB (%) | 5 | 5 | 95 | 95 | 5 | 5 |
| Detection: | 220 nm | | | | | | |
| MS Mode: | Positive | | | | | | |
| MS Range: | 100-1000 | | | | | | |

**4. Capture and fluorescence tracing the target proteins of the Tet probe**


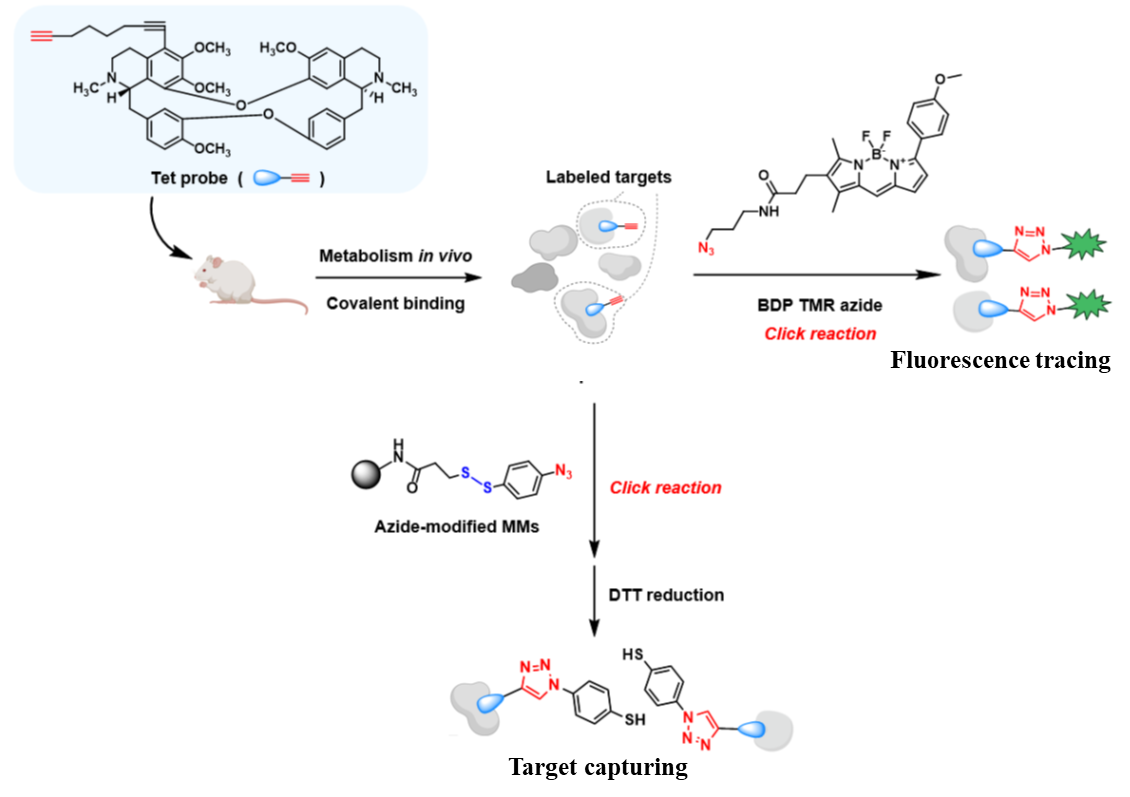


**Fig. S7 Schematic diagram of target capture and fluorescence tracing of the Tet probe *in vivo*.**

**5. Purification of human CYP11A1 and its mutant**

**
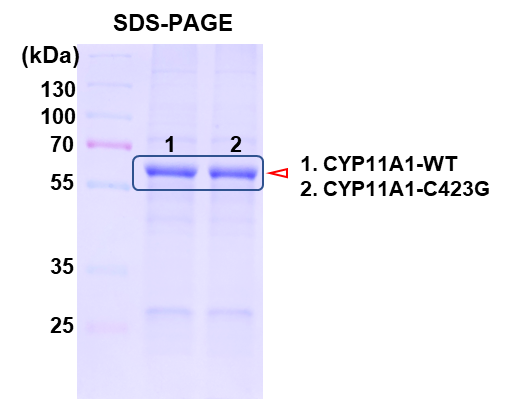
**

**Fig. S8. Coomassie brilliant blue staining of the purified recombinant protein CYP11A1 (about 60 KDa) and CYP11A1 mutant C423G (about 60 KDa).**

**6. The sequences information of *CYP11A1 siRNA* (product number: sc-41496)**

sc-41496: CYP11A1 siRNA (h) is a pool of 3 different siRNA duplexes:

sc-41496A:

•Sense: GCCUUUGAGUCCAUCACUAtt

•Antisense: UAGUGAUGGACUCAAAGGCtt

sc-41496B:

•Sense: GAAGUGUUCACCACGAUUAtt

•Antisense: UAAUCGUGGUGAACACUUCtt

sc-41496C:

•Sense: CUGCAGAGAUAUCUUGUAAtt

•Antisense: UUACAAGAUAUCUCUGCAGtt

All sequences are provided in 5′ → 3′ orientation.

**The origin western blot**

**Figure 1: The original image of Fig 2B in manuscript was presented as follow (the Western Blot was performed using 12.5% SDS-PAGE):**


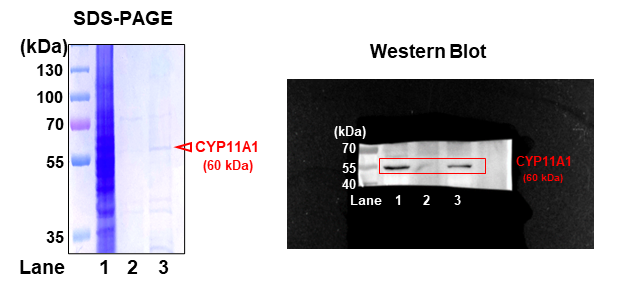


**Figure 2: The original image of Fig 3B for three repeats in manuscript was presented as follows (the Western Blot was performed using 10% SDS-PAGE):**


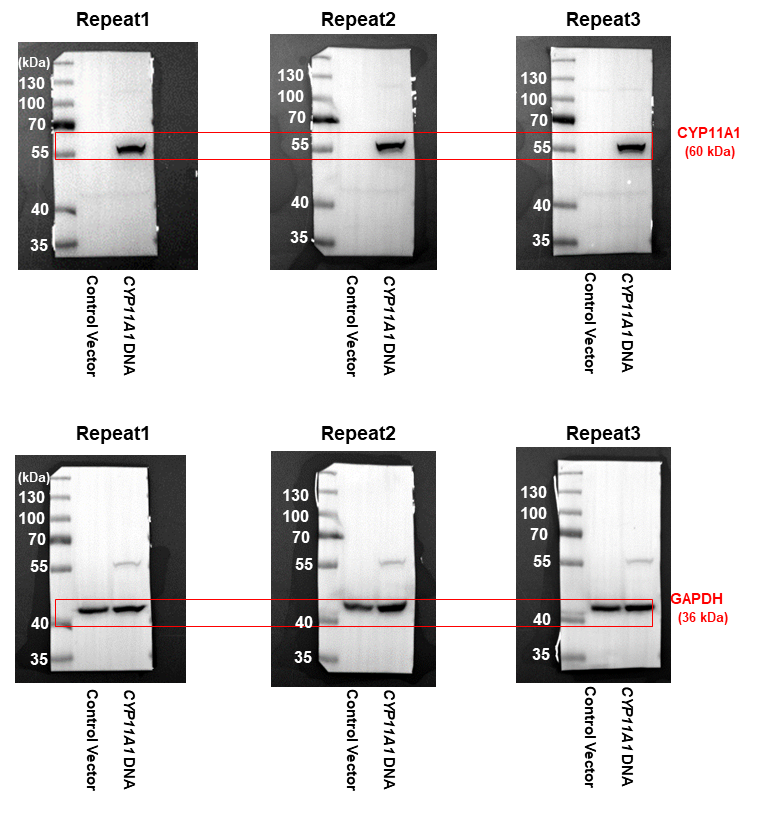


**Figure 3: The original image of Fig 4A in manuscript was presented as follow:**

**
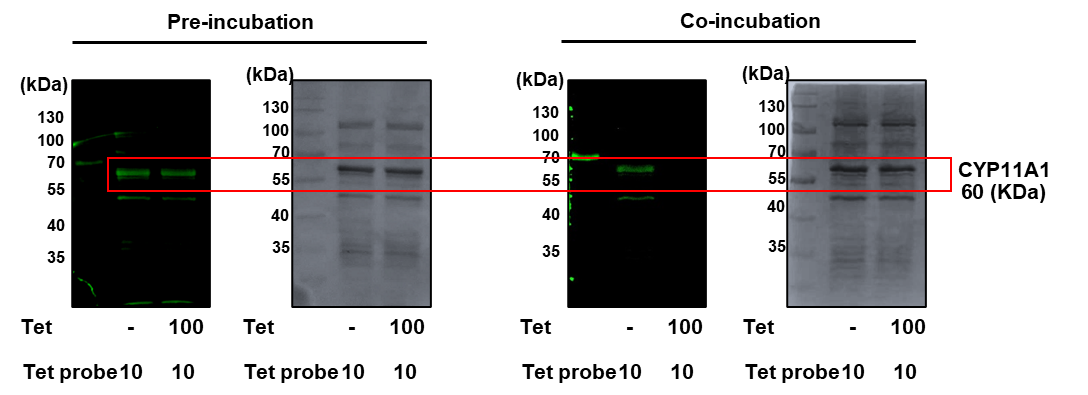
**

**Figure 4: The original image of Fig 4E in manuscript was presented as follow:**


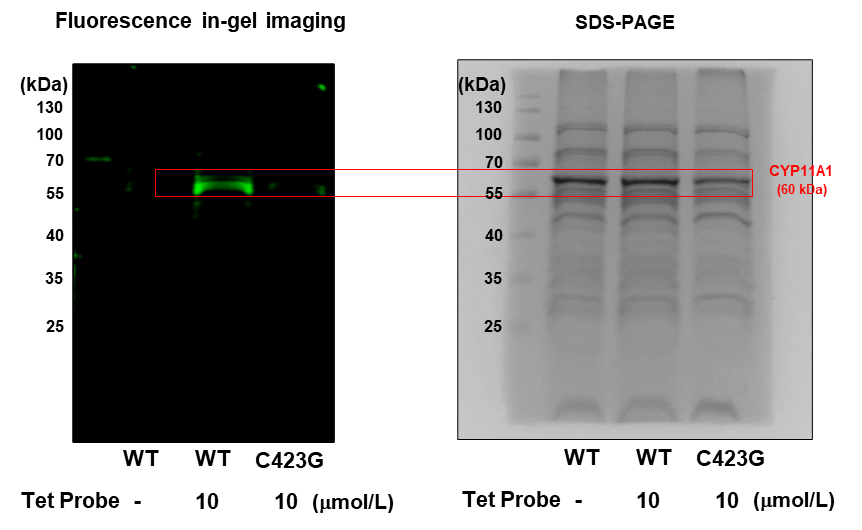

Supplement: Supplementary file 2 [file DataSheet1.docx]
